# Speeded saccadic and manual visuo-motor decisions: Distinct processes but same principles

**DOI:** 10.1016/j.cogpsych.2017.02.002

**Published:** 2017-05

**Authors:** Aline Bompas, Craig Hedge, Petroc Sumner

**Affiliations:** a CUBRIC – School of Psychology, Cardiff University, Cardiff CF10 3AT, Wales, United Kingdom; b INSERM, U1028, CNRS, UMR5292, Lyon Neuroscience Research Center, Brain Dynamics and Cognition Team, Lyon F-69000, France

**Keywords:** Action selection, Modelling, Response modalities, Competition, Reaction time

## Abstract

Action decisions are considered an emergent property of competitive response activations. As such, decision mechanisms are embedded in, and therefore may differ between, different response modalities. Despite this, the saccadic eye movement system is often promoted as a model for all decisions, especially in the fields of electrophysiology and modelling. Other research traditions predominantly use manual button presses, which have different response distribution profiles and are initiated by different brain areas. Here we tested whether core concepts of action selection models (decision and non-decision times, integration of automatic and selective inputs to threshold, interference across response options, noise, etc.) generalise from saccadic to manual domains. Using two diagnostic phenomena, the remote distractor effect (RDE) and ‘saccadic inhibition', we find that manual responses are also sensitive to the interference of visual distractors but to a lesser extent than saccades and during a shorter time window. A biologically-inspired model (DINASAUR, based on non-linear input dynamics) can account for both saccadic and manual response distributions and accuracy by simply adjusting the balance and relative timings of transient and sustained inputs, and increasing the mean and variance of non-decisional delays for manual responses. This is consistent with known neurophysiological and anatomical differences between saccadic and manual networks. Thus core decision principles appear to generalise across effectors, consistent with previous work, but we also conclude that key quantitative differences underlie apparent qualitative differences in the literature, such as effects being robustly reported in one modality and unreliable in another.

## Highlights

• Core architecture of visuo-motor selection model generalises across effectors.
• Hand and eyes show very different response times, but similar decision times.
• Longer non-decision time for visuo-manual responses accounts for longer response times.
• Stronger faster transient visual inputs for saccades account for different selection dynamics.

## 1 Introduction

The problem of how brains make decisions is central to cognitive psychology and neuroscience. Here we focus on rapid action selection between competing options signalled by simple clearly visible stimuli, such as making a hand response or an eye movement to a ‘target' stimulus in the face of alternative possibilities (distractors). The process of action selection contains many elements of broad interest to psychologists: the integration of volition (‘top-down' processes) with reflexes or stimulus-driven (‘bottom-up') processes; the idea of automatic, even unconscious, partial activation of response tendencies; the question of why we are variable and make errors even in the simplest tasks; the potential relationship between rapid decisions and personality traits or clinical symptoms such as impulsivity.

The key principle emerging from research on basic behavioural decisions is that sensory information and endogenous goals are thought to partially activate various response options, and the decision emerges through competition or interaction between the representations (populations of neurons) for each option (Kopecz, 1995, Leach and Carpenter, 2001, Usher and McClelland, 2001, Van Gisbergen et al., 1987). This conceptualisation thus assumes a strong coupling between decision processes and action planning. If this is true, action decisions are not domain general, but rather decisions about which button to press would be resolved within manual action areas (Donner, Siegel, Fries, & Engel, 2009), while decisions about where to look would be resolved in the eye movement network (Munoz and Wurtz, 1995, Purcell et al., 2010, Shadlen et al., 1996). The brain areas devoted to different modalities are organised in different ways and receive information at different rates from a different balance of pathways (Bompas & Sumner, 2008). This underlying anatomy and physiology has potentially important consequences for action decision dynamics throughout the process. The differences include the stage traditionally considered sensory, because of the different pathways feeding rapid action decision for different modalities.

The present article addresses whether there are key differences between manual and ocular decisions using behavioural and modelling approaches. This question may not be so critical for non-speeded abstract or difficult perceptual decisions, which may be less directly coupled to motor response representations. For example, for hard perceptual categorisation tasks, previous work has suggested that decisional mechanisms are the same regardless of response modality (Gomez, Ratcliff, & Childers, 2015) or are partly shared across modalities (Ho, Brown, & Serences, 2009). In contrast, the characteristics of individual response systems should be particularly key for rapid action selection based on relatively simple and suprathreshold stimuli, where competition between action options is likely to be the rate-limiting process and the main source of variance. The present article focuses on this category of task, which we anticipate are most sensitive to differences in properties and connectivity across motor systems. In this context, the word decision reflects the process resulting in the selection of action, while decision mechanisms reflects the necessary circuitry underlying this selection process.

### 1.1 Manual versus eye movement decisions

Although there are potentially key differences in sensorimotor dynamics for manual action and eye movements, most research traditions employ only one or the other while making general claims about action selection and decision. The majority of studies, and nearly all those involving patients or brain imaging, employ speeded manual button-press responses. These often rely on a variety of related paradigms to explore how response selection is influenced by mechanisms of attention, inhibition, expectation, reward, etc., often evolved from classic tasks such as Stroop, Simon, Eriksen flanker, Posner cueing, Stop-signal, and priming (Eimer and Schlaghecken, 2003, Eriksen and Eriksen, 1974, Logan et al., 1997, Posner and Cohen, 1980, Simon and Wolf, 1963, Stroop, 1935). Despite this variety, there is a common theme running through all these paradigms: in order to reveal the characteristics of underlying mechanisms, response options are put in competition with each other, and conditions that evoke response conflict are compared with conditions that do not.

While the mainstay of experimental psychology and human cognitive neuroscience has been manual button presses, a subset of human behavioural and modelling work and the majority of monkey neurophysiology studies on decision mechanisms have used saccadic eye movements (saccades). Drawing on both human and monkey data, functional models have been developed with explicit neurophysiological underpinning based on the known properties of neurons in saccade-related regions of the brain, such as the frontal eye field or the superior colliculus (Cutsuridis et al., 2007, Kopecz, 1995, Lo et al., 2009, Meeter et al., 2010, Purcell et al., 2010, Shadlen et al., 1996, Trappenberg et al., 2001). Nevertheless, many papers on saccade decisions are framed in terms of more general action decisions, claiming that saccades are simply a convenient model because the behavioural and neurophysiological details of saccades are relatively well understood. Indeed, the overarching principle of many of the tasks employed, such as antisaccades (Munoz & Everling, 2004) or distractor tasks (Walker, Kentridge, & Findlay, 1995), is competition and conflict between response options, just as in the manual tasks. However, the potential differences between saccades and other actions are rarely emphasised.

Before we can build a more fruitful bridge between the saccadic and manual literature, we first need to answer a basic question: how comparable is saccadic selection to manual selection? In rapid visual detection tasks, manual and saccadic responses have been reported to show different sensitivities to fixation stimulus offset (gap period, Iwasaki, 1990), stop signals (Boucher et al., 2007, Campbell, 2016, Logan and Irwin, 2000), inhibition of return (Briand et al., 2000, Sumner et al., 2004), visual distractors (McIntosh and Buonocore, 2012, Rafal et al., 1990, Ross and Ross, 1981, Sumner et al., 2002), Hick’s law (Kveraga, Boucher, & Hughes, 2002) and chromatic stimuli (Bompas & Sumner, 2008). Saccades and manual responses also have different operational constraints, different relative costs of making a mistake (Gilchrist, Heywood, & Findlay, 2003) and are differentially affected by alcohol (Campbell, Chambers, Allen, & Sumner, Registered report accepted in principle).

These differences are not surprising, considering that visually guided manual and saccadic responses are programmed through different neuronal networks (see Section 1.1.2), each with their own temporal dynamics fed by specific combinations of signals received from other parts of the brain. In addition to these general differences between effectors, the most common type of manual responses used in psychology and neuroscience – button presses in response to visual stimuli – present additional specificities. While saccades and reaching hand movements can be made anywhere in the (reasonably near) visual field, manual button presses are limited to a few options (typically two). Moreover, saccades are tightly linked to locations in retinotopic space, so that the onset of a peripheral stimulus will tend to trigger a single movement of both eyes to foveate it. Reaching movements are also non-arbitrarily linked to positions in space, but they are not programmed in retinotopic coordinates. Button presses are further removed from the visual signal, allowing more flexible mapping between retinal stimulation and motor activation and competition.

Despite these differences, commonalities are also apparent. For instance, both modalities appear to follow and violate Hick’s law, i.e. RT increases with the logarithm of the number of potential stimulus-response alternatives, but in response to converse sensory signals. Manual responses comply in responses to visual stimuli but not to tactile stimuli on the digits (Kornblum, Hasbroucq, & Osman, 1990). In contrast, saccadic responses violate Hick’s law in response to visual onsets but comply in response to less straightforward stimulus-response mappings (Kveraga et al., 2002). Similarly, in the stop signal task, where participants must inhibit a response to a target onset on rare trials where a “stop” signal appears, behaviour has been captured with the same simple model (the independent horse race model); only different parameters are used for manual and saccadic responses (Boucher et al., 2007, Logan and Irwin, 2000). These commonalities suggest that the differences observed across effectors could largely depend on the nature of the sensory inputs and their access to the different brain areas involved in action planning, rather than fundamental differences in the way decisions are taken across effectors. Therefore, although these previous studies suggest that action selections made via different effectors involve distinct processes, they could well share the same principles (Logan & Irwin, 2000). However, this conclusion may derive from tasks that happened not to tap the critical differences across effectors and could possibly be challenged by more sensitive tasks (see Section 1.2).

To address the question of how manual and saccadic visuo-motor decisions may functionally differ, we turn to a family of quantitative models. In these models, nodes representing each response possibility are activated in parallel by relevant stimulus information, essentially accumulating evidence for each response option (Boucher et al., 2007, Kopecz, 1995, Leach and Carpenter, 2001, Purcell et al., 2010, Usher and McClelland, 2001, Wilimzig et al., 2006). For models with multiple nodes organised in maps, nodes coding for spatially similar actions activate each other, while distant locations compete with each other (‘interactive competitive accumulation') through mutual or feed-forward inhibition. The first node to reach a defined activation threshold wins the decision, and that response option is carried through to execution. The exact implementation of these models and their neurophysiological underpinning is still debated (see Section 8.7 for discussion), but their strength lies in explicitly capturing key and dominant ideas in the decision literature (Kopecz, 1995, Usher and McClelland, 2001, Wilimzig et al., 2006). Our aim here is to question how transferable these concepts are across action modalities.

From the elements introduced above, one can already conclude that a highly detailed model for saccade selection is unlikely to directly apply for button presses. However, without a common generative model, it is difficult to go beyond superficial comparisons of behaviours across modalities (comparing for instance *descriptive* variables related to reaction time distributions or error rates). In order to assess the similarity and specificities of the underlying mechanisms in both modalities, we need to allow a single model architecture to account *overall* (if not in all details) for the constraints of both modalities. At the same time, this model needs to be detailed enough to be able to reveal these specificities if they exist. The present article proposes such model and assesses three fundamental factors: (1) decision vs non-decision time and variability; (2) the balance and timing of input signals; (3) winner-takes-all behaviour and lateral inhibition.

#### 1.1.1 Decision vs non-decision time and variability

In rapid action selection tasks, temporal differences are immediately apparent between latency distributions for different modalities. Saccadic responses are faster, and often show an even faster “shoulder” on the left of the main mode of the distribution. This fast volley starts around 70 ms and is not reducible to anticipatory saccades (Fig.2A, compare the correct and incorrect responses, i.e. thick and thin grey lines), it is therefore indicative of the shortest delay required for visual input to drive or interfere with saccade initiation. Manual responses usually present a single, later, less skewed and wider mode. Despite this clear difference being long and widely known, the most fundamental question remains unanswered: whether it represents a difference in the decision process itself or non-decisional input and output delays, or both.

At least some difference in non-decision time is expected but has never been precisely estimated. Saccadic RTs are measured from the very beginning of the saccade and can be accurately detected using a velocity threshold. Saccadic motor output time in the monkey is consensually estimated to be around 20 ms, based on the minimal delay between electrical stimulation in the SC and saccade onset (Munoz and Wurtz, 1993, Smit and van Gisbergen, 1989). In contrast, button press RTs are detected only when some movement has been executed and the exact time taken by the button press itself may vary across devices and participants, as well as across trials. Manual motor output time between primary motor cortex activity and single finger movement has been recently estimated to be around 85 ms using intra-cranial EEG in human (Miller, Zanos, Fetz, den Nijs, & Ojemann, 2009), based on the latency of the peak correlation between finger displacement and activity recorded at the corresponding cortical surface. On the other hand, visually evoked responses from task-relevant stimuli in humans have been reported in the shoulder muscles from as early as 75 ms post-stimulus (Pruszynski et al., 2010).

For difficult perceptual discrimination, Ho et al. (2009) used the linear ballistic accumulator model (Brown & Heathcote, 2005) to compare manual and saccadic responses and concluded that the difference can be attributed to both longer non-decision and longer decision times for manual responses. In a demanding letter discrimination task, Gomez et al. (2015) used the diffusion model (Ratcliff & Rouder, 1998) and reached a similar conclusion. However, reaction times in hard discrimation tasks were substantially longer than those observed in speeded action selection tasks. It is therefore unclear whether the conclusions from this previous work generalise to rapid action selection to clearly discriminable stimuli. Besides, in these previous studies, non-decision times were modelled either as constant across trials (Ho et al., 2009) or as a uniform distribution (Gomez et al., 2015), while recent work has highlighted the importance of correctly identifying the non-decision time distribution in order to correctly infer the decision process (Verdonck & Tuerlinckx, 2016).

In previous work on saccadic eye movements, we described how the interference from irrelevant visual distractors during saccade planning (see Section 1.2.1) was able to provide a precise estimate of the saccadic non-decision time (Bompas & Sumner, 2011). One main purpose of the present paper is to apply the same design and logic for manual responses, and thus to infer whether saccadic and manual decision time is similar or dissimilar, and relatedly, whether the extra variance in manual responses comes from within the decision process or is introduced during non-decisional stages. To anticipate, our results and simulations will suggest that:

(1) The extra delay and variance in manual responses are mainly due to differences *outside* the selection process (input and output delays), rather than longer decisional time.
(2) Manual decision times are likely to be similar to saccadic ones.
(3) Manual output times are well captured by a gamma distribution.

#### 1.1.2 The balance and timing of input signals

Visually guided saccadic and manual responses in primates rely at least partly on different anatomical pathways. Saccades are produced via the brainstem, which receives direct projections from the superior colliculus (SC) and the frontal eye fields (FEF), as well as indirect projections from the parietal and visual cortices (Liversedge et al., 2011, Schiller et al., 1979). The superficial layers of the SC receive strong and very rapid visual inputs both directly from the retina and from primary visual cortex (Sparks, 2002, White and Munoz, 2011). With minimal delay, the first volleys occur in FEF and deeper layers of SC. These initial inputs are non-selective for relevant target properties, but tend to strongly drive many cells in monkey neurophysiology studies (Dorris, Olivier, & Munoz, 2007). The first signs of selective modulation of inputs to FEF and SC occur at about 50 ms (Munoz and Wurtz, 1995, Schmolesky et al., 1998). Although the SC also plays a role in other motor responses, including head (Corneil, Olivier, & Munoz, 2002) and arm movements (Werner, Dannenberg, & Hoffmann, 1997), the programming of manual responses is thought to mainly involve the primary and supplementary motor cortices. Sensitivity to visual stimuli in some neurons within these cortical areas has been reported, but is commonly agreed to be less pronounced than for saccades, and appears mostly in response to motion (Rizzolatti et al., 1981, Wannier et al., 1989) or task-relevant stimuli (Pruszynski et al., 2010). It would thus be expected that manual responses should be less sensitive to fast unselective visual inputs and rely comparatively more on later selective signals.

The various information pathways into the action selection networks (Bompas et al., 2008, Bompas and Sumner, 2008, Bompas and Sumner, 2009a, Sumner et al., 2006, White and Munoz, 2011) are normally simplified for models either into a single source of evidence/activation (Brown and Heathcote, 2005, Carpenter and Williams, 1995, Logan et al., 2015) or into two types: non-selective (‘exogenous') signals arriving at short delay and selective (‘endogenous') signals arriving later (Bompas and Sumner, 2011, Trappenberg et al., 2001). We took the later approach. To anticipate, our data and simulations suggest that saccadic responses are subject to a relatively stronger and faster influence of exogenous signals than are manual responses, and weaker influence from endogenous signals. These differences, occurring within the action selection process, support the existence of modality-specific decision stages, while still being consistent with the existence of an *additional* amodal stage (Ho et al., 2009). Adjusting the balance and timing of input signals to the competitive decision process, together with assuming extra non-decision time and variance for manual responses (see Section 1.1.1), was sufficient to accommodate the patterns of results in both modalities.

#### 1.1.3 Winner-takes-all behaviour and lateral inhibition

While both eyes (usually) move together, and thus only one response can be expressed at a time, such a strong, hard-wired constraint does not exist for manual responses: left and right button presses are mutually exclusive only if the task says so, and low compliance (or different instructions) could lead to both buttons being pressed at the same time (Schlaghecken, Klapp, & Maylor, 2009). On the one hand, the scarcity of trials where both buttons are pressed together strongly suggests that, from a computational point of view, alternative action plans behave as mutually exclusive in this kind of task setting. On the other hand, the fact that these dual button press trials still occur sometimes could mean that the selection of a manual response does not require a strong “winner-takes-all” process, thus suggesting possibly weaker mutual (lateral) inhibition between the neurons coding alternative responses than for saccadic responses. To anticipate, although our modelling does not exclude this possibility, we find that reduced mutual inhibition in manual action selection compared to saccades is neither necessary nor sufficient (or even clearly helpful) to account for the different behavioural pattern we observe between manual and saccade responses.

### 1.2 A basic phenomenon for investigating competition dynamics

#### 1.2.1 RDE and saccadic inhibition

In order to directly compare manual and saccadic rapid action selection and assess the three fundamental factors outlined above, we employ the simplest form of conflict task. Simple tasks that depend critically on rapid response selection ought to be most dependent on modality-specific processes, so a simple sensorimotor task is the strongest test for generalizability. A ‘target' stimulus is presented either with or without an accompanying irrelevant ‘distractor' stimulus. In saccades, such a paradigm yields two related phenomena, known as the ‘remote distractor effect' (RDE) and ‘saccadic inhibition' (Bompas and Sumner, 2015, Buonocore and McIntosh, 2008, Reingold and Stampe, 2002, Walker and Benson, 2013). In the RDE, the presentation of a distractor in a location remote from a saccade target, before or together with target onset, delays the whole latency distribution of saccades to that target and may increase errors (Fig.2A). ‘Saccadic inhibition' refers to a characteristic effect on the saccadic latency distribution when distractors appear *after* the saccade target. There is a dip in the number of saccades initiated around 70–100 ms after distractor onset (Fig.2B). This phenomenon was first reported in reading studies (Reingold and Stampe, 1999, Reingold and Stampe, 2000, Reingold and Stampe, 2003, Reingold and Stampe, 2004), and then shown to generalise to other eye movement tasks (Buonocore and McIntosh, 2008, Buonocore and McIntosh, 2012, Edelman and Xu, 2009, Reingold and Stampe, 2002).

Although it remains possible that the RDE and SI are produced partly by distinct mechanisms (Walker & Benson, 2013, and see Section 6.5; 2015), a parsimonious hypothesis is to simply view the shift in latency distribution for simultaneous distractors as a ‘dip' on the leading edge of the distribution – to view the RDE and saccadic inhibition as two ways of measuring the same fundamental interference process (Buonocore and McIntosh, 2008, McIntosh and Buonocore, 2014). Crucially, both phenomena can be captured using a single model involving rapid visual input to, and lateral inhibitory connections within, a saccade motor map, without changing the values of any parameter (Bompas and Sumner, 2011, Bompas and Sumner, 2015).

#### 1.2.2 A key difference between saccades and manual responses?

Taken together, the effects of simultaneous and late distractors on latency distributions of correct responses and errors impose strong constraints on action selection models. However, the few studies that have tested the RDE for manual responses have not found it (McIntosh and Buonocore, 2012, Rafal et al., 1990, Ross and Ross, 1981, Sumner et al., 2002). Most recently McIntosh and Buonocore (2012) reported three experiments comparing saccades to manual reaching, all of which found clear saccadic RDEs but no (or tiny) manual RDEs. From this evidence we might surmise that manual latency distributions are immune to interference from irrelevant distractors. If true, this has fundamental consequences for models of speeded manual decisions, suggesting an absence either of automatic visual signals to manual decision areas, or of mutual inhibition/subtractive inputs. All models that contain these basic features will by necessity produce an RDE.

On the other hand, manual response times are well known to be susceptible to interference from irrelevant stimuli in paradigms such as masked priming, the Eriksen flanker task, or the Simon task. All these tasks provide evidence of automatic and rapid partial activation of, and functional inhibition between, alternative manual responses. From this evidence we might predict that manual responses ought to show a similar effect to the RDE and saccadic inhibition and that previous null results are the consequence of a poor choice of parameters or insufficient statistical power (see Section 8.1 in Discussion).

To anticipate, we observed clear effects on manual latency distributions from simultaneous and slightly delayed (20 or 40 ms) distractors, but no clear effect at longer SOAs. These behavioural similarities justify our attempt to capture both modalities within a single model. However the amplitude and timing dependencies of these effects were also clearly different from those on saccades, setting up a challenge for any unified model to overcome.

### 1.3 Overview of the present article

To discover whether manual responses show the signs of distractor interference, we first investigated a large range of relative timing between target and distractor (Experiment 1, stimulus onset asynchrony, SOA from 0, 50, 100, 150 and 200 ms, Sections 2, 3). Manual responses clearly proved sensitive to simultaneous visual distractors but, contrary to saccadic responses from previous work (Bompas & Sumner, 2011), interference was not present at SOA 50 ms or beyond. Furthermore, this interference appeared much later within the latency distribution, in line with longer overall latency of manual responses.

To test whether a single model architecture could capture both modalities, we introduce a simplified 2-node version of previously published DINASAUR model, each node coding for one response option (left or right). We first adjusted the parameters for saccades (variant S1) and then tested two extreme hypotheses of how manual decision and non-decision times could compare to saccadic ones (Section 4):

– **M1** assumes manual responses share the exact same decision process as saccades, while the extra delay compared to saccades entirely reflects the longer and more variable non-decision times.
– **M2** assumes manual response non-decision time is the minimum delay suggested by electrophysiology, and the remainder of the extra delay compared to saccades reflects longer decision times.

To anticipate, we find that variant M1 provided a much better match to our data, and can be further improved by fine-tuning the balance and timings of inputs (i.e. the decision process). To help constrain these changes, we acquired a second independent dataset (Experiment 2), using the same participants for the saccadic and manual versions of the same task, at SOAs likely to show interference – 0, 20 and 40 ms (Section 5). Model parameters were then adjusted in two steps (Section 6), inspired by neurophysiological indications (Section 1.1.2):

– **M3** makes similar assumptions as M1 regarding non-decision time and additionally the decision process relies less heavily on transient exogenous signals and more on sustained selective (endogenous) signals (i.e. the *amplitude* of transient signals is reduced).
– **M4** is the same as M3 but the *latency* of exogenous inputs is also delayed for manual compared to saccadic decisions.

We conclude that M3 improves on variant M1, and M4 further improves on M3, providing excellent match to the observed behaviour. Last, we show that a change in mutual inhibition is neither sufficient nor even helpful to tackle the relationship between saccades and manual responses (Section 7).

## 2 Material and methods of Experiment 1

### 2.1 Participants

Three observers participated. Observer 1 was author AB, while the other two participated in exchange for a small monetary award. All had normal vision.

### 2.2 Stimuli and procedure

We used published saccadic data from Bompas and Sumner (2011), which we directly compared with new data from new participants using the same protocol but with button press responses instead of saccades. Targets could appear randomly on the left or right of fixation, and small distractors appeared in the alternative target location (i.e. a task-relevant location) randomly on 5 out of 6 trials (83%). Stimuli were displayed binocularly with 72 cm viewing distance on a Sony Trinitron 19 in. GDM-F400T9 monitor, driven by a Cambridge Research Systems (CRS) ViSaGe graphics board at 100 Hz, calibrated with a CRS ColorCal and associated software. Participants were instructed to fixate and fixation was monitored using the CRS high-speed (250 Hz) video eye-tracker mounted on a combined chin and headrest. Manual reaction times were acquired via the CRS CB6 response box, that directly interfaces with the ViSaGe via an infra-red link and provides accurate high-resolution time measurement. The fixation point was a small light grey square (32 cd·m^−2^ occupying 0.1 × 0.1 deg^2^), and appeared at the start of the trial, on a grey background (25 cd·m^−2^, MacLeod-Boynton coordinates, MLB, 0.643, 0.021). A fixed delay (700 ms) later, the target stimulus, a small black square (10 cd·m^−2^, occupying 0.25 × 0.25 deg^2^), appeared randomly on the left or on the right of fixation (8 deg). Observers were instructed to respond rapidly to the target direction by pressing the left or right button (using their left and right index finger), ignoring any other stimuli. Participants were instructed to respond ‘as quickly as possible whilst minimising errors'. Fixation and target stimuli extinguished together after 300 ms, and fixation reappeared 500 ms later to begin the next trial. Distractors were grey squares (1 deg^2^, 30 cd·m^−2^), and appeared opposite the target for 50 ms, also centred at 8 deg eccentricity. These were presented with SOAs of 0, 50, 100, 150 and 200 ms, in order to span across the entire latency distribution, randomly shuffled in with the no-distractor trials. There were 250 trials per condition per observer (500 trials per condition when pooling left and right targets), split into 6 blocks of 15 min each.

### 2.3 Reaction time analysis

To compare baseline (no-distractor) RT distributions across modalities, we searched to characterise the delay distribution that should be added to saccadic RTs to make them similar to manual RTs. Note that this approach does not provide straightaway the distribution of non-decision times in manual responses, but provides a good approximation under the assumption that the decision times are similar across modalities and that saccadic output times have very little variance. In order to do this, we searched for the noise distribution which, when convolved with the saccadic baseline RT distribution, would minimise the distance to the manual baseline RT distribution. We considered 3 types of noise – uniform, Gaussian and gamma – and fitted the parameters (2 for uniform and Gaussian, 3 for gamma) providing closest match. We repeated this procedure 10 times and compared the Kolmogorov-Smirnov (KS) distance between the raw manual RTs and the raw saccadic RTs to which noise randomly drawn from each distribution had been added.

Manual latency distributions were obtained with a bin size of 4 ms (for consistency with the saccadic analysis using an eye-tracker at 250 Hz). To get robust estimates of dip timings, all distributions were smoothed using the same Gaussian kernel with 7 ms window and 3 ms standard deviation and interpolated to obtain 1 ms precision (previous work using twice more trials per condition used 5 and 1). This smoothing is necessary to get meaningful estimates from noisy data but it tends to underestimate estimates of dip onset (T_0_) and minimum RT by about 7 ms. Because smoothing is consistently applied through all conditions, we did not correct for this.

To evaluate the amplitude and timing of dips, we calculated the ‘distraction ratio’ for each time-point (shown in Fig.2E–H), following Reingold and Stampe (2004). In order to better characterise the interference from distractor, it is often useful to pool data across SOA conditions, in which case the distractor-to-response time is used to calculate the distraction ratio, rather than the classical RT from target onset. Indeed, the timing of any inhibition effect on the latency distribution is expected to be time-locked to when the distractor appeared (see Bompas & Sumner, 2011, for details). The distraction ratio, at each time bin, is defined as the reduction in the number of correct responses in the distractor-present distribution (*N_d_*) from the baseline (*N_b_*), relative to the number in the baseline distribution (i.e. (*N_b_* − *N_d_*)/*N_b_*). This ratio is unstable for very small *N_b_*. When high numbers of trials per curve are available, we required *N_b_* > 10 as a criterion for calculating the ratio (Fig.2E–F and G–H). In Supplementary Fig. 1 we required N_b_ > 2 due to lower numbers per curve. A dip was detected if the maximum distraction ratio reached a value of at least 16% with a (*N_b_* − *N_d_*) difference of at least 2. Dip onset (*T*_0_) was estimated by going backward in time within the distribution from the maximum of the ratio (*T_Max_*) until the ratio falls below 2% or the (*N_b_* − *N_d_*) difference falls below 1. Employing the ratio rather than the simple difference between the distractor and no distractor conditions ensures that dip parameters are independent of when the dip occurs within the distribution, i.e. whether it occurs when there are many or few responses in the baseline distribution. Positive deflections show the inhibition effect, and subsequent negative deflections show the recovery phase.

Minimum RT was defined for the baseline condition as the shortest RT with *N_b_* > 1 (at least one saccade after smoothing) and the difference between the number of correct and incorrect saccades (*N_b_* − *N_bi_*) > 1. Using a large number of trials per condition in conjunction with smoothing guaranties that this estimate is robust and indicates indeed the earliest responses that include *some* visual input, rather than pure anticipations. Note that this does not mean that *all* RTs equal or longer than this minimum are visually-driven and not anticipatory.

To assess the reliability of peaks and troughs of the distraction ratio, bootstrapping was performed on individual data. Original data for each condition and participant was subsampled with replacement to obtain 1000 surrogate datasets containing the same number of trials as the original condition (Supp. Fig. 2).

## 3 Behavioural results – Experiment 1

**Fig. 1:**
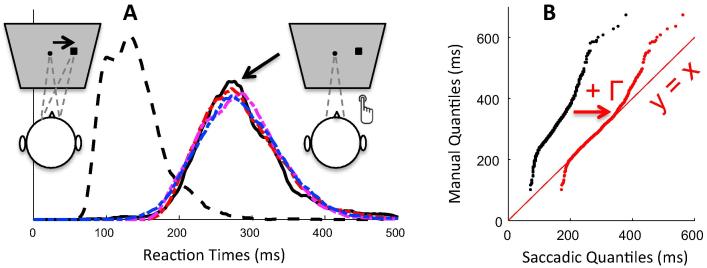
Baseline (no distractor) RTs across modalities. (A) RT distributions of observed saccadic (dashed black line) and manual (full black line) correct RTs pooled across observers. These are shown together with the best attempts at bridging the gap across modalities by adding uniform (pink), Gaussian (blue) or gamma (red) noise plus a fixed time delay to saccadic RTs. All three types of noise provide reasonable matches visually; the gamma distribution (red) provides the best match (see Supp Fig. 1). (B) Q-Q plot of the same data, before (black points) and after (red) adding gamma noise to saccadic RTs. Although the Q-Q plot remains highly non-linear, adding a delay of 79 ms and gamma noise (λ = 3, *k* = 23) allows it to regress on the identity line for a substantial part of the distribution. (For interpretation of the references to colour in this figure legend, the reader is referred to the web version of this article.)

**Fig. 2:**
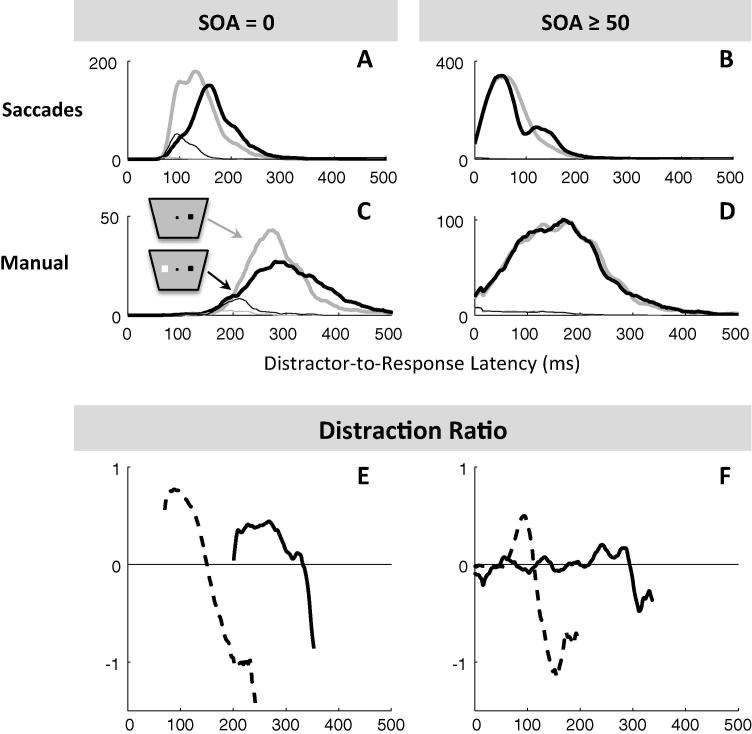
Comparison of the effect of simultaneous (left column) and late distractors (right column) on saccadic (A-B) and manual (C-D) reaction times, pooled across observers. The distributions are time-locked to distractor onset in order to combine data across distractor SOAs. (A–D) Thick black and grey lines correspond to correct responses, in the presence and absence, respectively, of a distractor. Thin lines show incorrect responses. Although manual response distributions are later than for saccades, the effect of simultaneous distractors is broadly the same (compare A and C), though later and smaller on average. See Fig. 3 for individual distributions. (E and F) Distraction ratios for saccades (dashed), manual responses (full), i.e. proportional change of responses in the distractor-present distribution relative to the number in the baseline distribution. For late distractors, the effect on saccades is a dip in the distribution time-locked to distractor onset (B, F) while any such effect is unclear for manual responses (D), although the distraction ratio (F) does show a small rise between 200 and 300 ms. See Supplementary Fig. 2 for individual ratios.

**Fig. 3:**
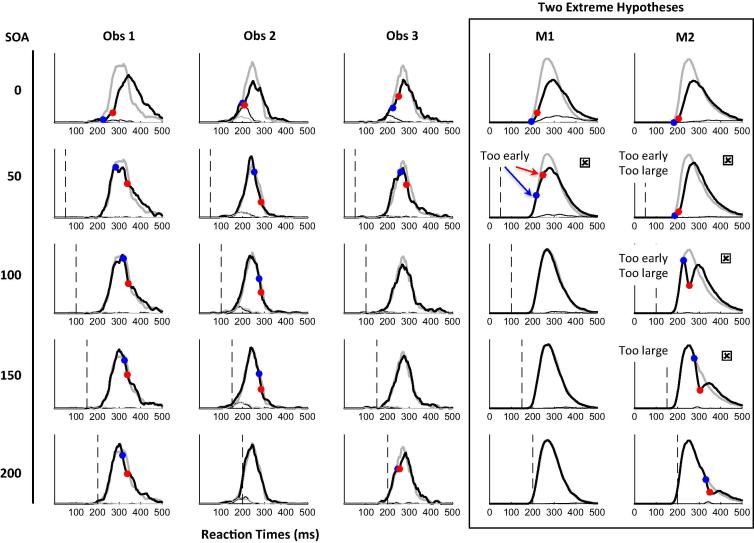
Manual reaction time distributions in the absence (grey lines) and presence (black lines) of a distractor for each observer in Experiment 1 (columns 1–3) as well as simulated from two hypothetical manual variants of our model (see Section 4.3). Each row represents a different stimulus onset asynchrony (SOA) between target and distractor stimuli (vertical dashed line indicates distractor onset). Thick and thin lines correspond to correct and incorrect responses respectively. Blue and red dots indicate the onset (T_0_) and maximum (T_Max_) of dips, when these are detected. All model distributions, T_0_ and T_Max_ estimates were obtained by averaging across 10 independent simulations. In contrast to saccades, manual responses show no clear dips in the distribution for distractors appearing 50 ms or more after the target. Variant M1 (longer output time than saccades) correctly captures this absence for SOAs 100 ms and longer but incorrectly predicts an RT cost at SOA 50. In contrast M2 (longer decision time than saccades; see Section 4.3 for details and explanation) fails at all SOAs. (For interpretation of the references to colour in this figure legend, the reader is referred to the web version of this article.)

Table 1 and Fig. 1, Fig. 2 provide an overview of baseline RT and distraction effects across modalities, combining the saccadic data from the main experiment in Bompas and Sumner (2011) and current Experiment 1. Individual distributions and dip timing estimates are provided in Fig. 3 and Supplementary Fig. 2.

### 3.1 Baseline RTs across modalities

Fig.1A offers a comparison of baseline (no distractor) RT distributions across modalities. As expected in a fast action selection task, manual RTs are clearly longer than saccadic RTs. Though more variable, their distributions also tend to be less skewed. We then characterised the approximate delay distribution that should be added to saccadic RTs to make them similar to manual RTs (see Section 2.3).

Each type of distribution (uniform, Gaussian or gamma) allowed a good match visually (Fig.1A), with the gamma distribution offering a better match consistently across 10 repetitions (Supplementary Fig. 1 shows the KS statistics for best fits across repetitions). Best parameters for the gamma distribution were a shape of λ = 3 and a scale *k* = 23 on top of an RT offset of 79. Applying such gamma shaped delay to the observed saccadic baseline data also ensured a close match to a y = x regression line in the Q-Q plot from the pooled data across observers (Fig.1B), further showing it captures well extra delay and variance between modalities. Importantly though, Fig.1B clearly shows that the Q-Q plot is not a straight line, with or without noise, which suggests that differences across modalities are not exclusively reducible to differences in non-decision time or variance.

### 3.2 Distraction effects in manual responses

Fig. 2 pools across observers in order to visualise the main findings. For simultaneous distractors, (Fig.2A and C), the inhibition effect is clear for both modalities (all KS tests comparing the distractor to the baseline conditions pooled across observers have an associated p < 10^−6^). The distraction ratio plots (Fig.2E and Supp. Fig. 2B) show that the inhibition effect for manual responses is delayed by about 140 ms compared to that for saccades. For late distractors (SOA 50–200) in the manual modality there is no clear distraction effect (Fig.1D, p > 0.6), in stark contrast to the clear dip in the distribution for saccadic responses (Fig.1B, p < 10^−6^). As such, while the saccadic distraction ratio shows a clear rise beginning at 70 ms (Fig.1E), the manual distraction ratio hardly departs from zero (Fig.1F). There is however a small effect about 210–290 ms after distractor onset, i.e. at the delay expected from the results at SOA 0 (Fig.1F).

All the aspects highlighted in Fig. 2 were also present at the individual level (see Fig. 3 and Supplementary Fig. 2). In the manual condition at SOA 0, all participants showed clear evidence of an inhibition effect (Fig. 3, all p < 0.01 for the individual KS test), starting shortly after 200 ms with a maximum around 250 ms after distractor onsets. Another signature of interference from simultaneous distractors on manual decisions was the increased error rates in all three observers (from 4% to 12% on average, p < 0.05, KS test on error distributions pooled across the three observers between the no distractor and SOA 0 conditions, compare the thin grey and black lines in Fig. 2C).

For longer SOAs, there was no clear evidence of interference from irrelevant stimuli (all p > 0.05, if an effect is there, it is hard to distinguish it from random fluctuations of the distributions), either on reaction times or on error rates. RT costs (mean latency difference between the distractor present and absent conditions) at SOAs 50 and beyond were all negligible (between −5 and 5 ms, 0 ms on average). Nevertheless, our detection algorithm still detects very small dips for some participants and some SOAs, the timing of which seems to be locked on distractor onset, thus suggesting some form of interference (see Fig. 5 and Supplementary Fig. 3). However, the absence of RT cost suggests that the small number of affected responses is not delayed, but rather omitted or directed to the distractor.

Thus, although inhibition is present for SOA 0 in all three subjects, it is almost absent at longer SOAs. This clearly contrasts with the results in the saccadic version of the same task (see Fig. 1 and Bompas & Sumner, 2011), where late distractors always showed signs of interference as long as there were enough trials in the latency distribution to observe their effect (all p < 10^−6^ at all SOAs for slower observers and up to SOA 60 for faster observers). Although our previous article did not include SOAs 50 and 100 ms in the saccadic task (we used 0, 20, 40, 60 and 80 ms), it was clear that dips in the latency distribution would have been observed if we had included these conditions.

## 4 Modelling results: decision versus non-decision time

The presence of clear interference from simultaneous distractors on manual reaction times and accuracy suggests similarities with the saccadic sensorimotor system: visual signals also automatically interfere with manual responses, and target and distractor activities appear mutually inhibitory. This suggests that both systems could be modelled with similar architectures. On the other hand, the two systems also show clear differences: in addition to the basic differences between manual and saccadic latency distributions, saccades show a robust inhibition effect for later distractors, while it is not clearly visible for manual responses as soon as distractors are delayed by only 50 ms. A first aim here is to assess whether these behavioural differences are best accounted for by decisional or non decisional differences between modalities. With this in mind, we now introduce a relatively simple model able to generalise across modalities.

### 4.1 Previous modelling of saccadic behaviour with the 200N-DINASAUR

In previous work (Bompas & Sumner, 2011), we described a competitive leaky accumulator with highly non-linear dynamics based on a model derived from neurophysiological recordings in the superior colliculus (Trappenberg et al., 2001), simulating a one dimensional saccade map with 200 nodes as a simplified representation of left and right superior colliculi. This model is strongly inspired from previous modelling work (Usher & McClelland, 2001) but benefits from two separate inputs representing the transient automatic and sustained selective input signals measured neurophysiologically (Dorris et al., 2007, Schall et al., 1995). The transient input proved crucial in capturing the effect of visual distractors on saccades, and we call this type of neuronally inspired model ‘dual input neural accumulation with selective and automatic rise’ (DINASAUR, Bompas & Sumner, 2011). It is important to note that the model was not designed to simulate saccadic inhibition – the phenomenon was unknown to the original designers (Trappenberg et al., 2001). Rather, it was designed to emulate the SC in a relatively simple way, and to capture the effect of distractors and fixation stimuli occurring before target stimuli. However, in distilling key properties of SC neuronal activity into the model, it turns out that DINASAUR automatically produces saccadic inhibition (Bompas & Sumner, 2011).

Before the target comes on, the activity within the SC map is kept under control by the high activity at fixation, inhibiting the rest of the map via lateral interactions and preventing unwanted anticipatory saccades. At target onset, nodes representing responses around target location are activated, via transient and then selective signals, and start inhibiting fixation nodes, until one peripheral node reaches threshold and a saccade can be triggered to the corresponding location. When a distractor is present, nodes representing the distractor also get activated by transient exogenous signals, though not by later selective signals. This has two consequences: (1) sometimes distractor activity can reach threshold first, leading to saccades being directed to the distractors (errors), (2) the target nodes now also receive inhibition from distractor nodes and thus reach threshold later than they would have done without the distractor present, so the latency is longer. The feature that allows it to capture such interference from a distractor even at long SOAs is the sharpness of the exogenous signal, which unlike a linear input, can transiently overcome mutual inhibition even when the target node is already highly active, as anticipated in early studies describing saccadic inhibition (Reingold and Stampe, 2000, Reingold and Stampe, 2002).

### 4.2 2N-DINASAUR

**Fig. 4:**
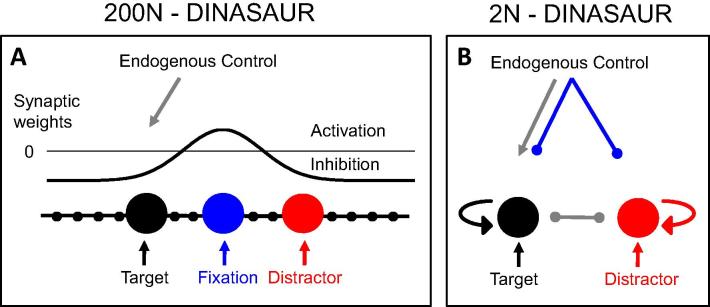
Architecture of the 200N (A) and 2N-DINASAUR (B) models. (A) The original version of DINASAUR with 200 nodes, inspired from Trappenberg et al. (2001) and previously described in Bompas and Sumner (2011). (B) The 2-node version of the model introduced here allows for a fair comparison with manual responses. It also has mutual inhibition, self-excitation and leakage, an endogenous signal that favours the target. It no longer contains a fixation node to keep neural activity stable before stimuli appear; instead this role is performed by common endogenous inhibition.

*Rationale.* The original DINASAUR model (Section 4.1) simulates the horizontal midline with 200 nodes (including a fixation node in the middle). This is because, in principle, saccades could be made to any location during the task. However, for button presses, only two options were available. Therefore, we made a version of the model with only two nodes, 2N-DINASAUR (Fig. 4) which, while losing the ability to simulate well-known effects of the saccadic literature (express saccades, strong gap effect, spatial error of saccade endpoint), has three advantages for us: it is directly transferable between manual and saccadic responses; it does not contain spatial activation profiles, and thus there is no need to constrain them (or assume they are the same as in monkey); it is computationally simpler, and thus slightly faster for simulations.

*Dynamics.* The 2N version keeps the same dynamics as the 200N version. The average spiking rate *A_i_* of neuron *i* is a logistic function of its internal state *u_i_* and a fixed steepness parameter *β*

$$A_{i}(t)=1/(1+\exp(-\beta u_{i}(i)))$$

The internal state *u_i_* varies across time *t* according to the following equation

$$\tau \frac{{\mathrm{du}}_{i}(t)}{\mathrm{dt}}=-u_{i}(t)-u_{0}+\mathrm{Act}\cdot A_{i}(t)+\mathrm{Inh}\cdot A(t)+I_{i}^{\mathrm{exo}}(t)+I_{i}^{\mathrm{endo}}(t)+a_{\eta }\eta (t)$$

where the essential features of the model are the separate transient (exogenous) and sustained (endogenous) input signals (*I^exo^* and *I^endo^*), and the influence of lateral inhibition from the activity *A_j_* of the other neuron *j* and the self-excitation proportional to the activity *A_i_* of neuron *i*. The model also includes leakage (−*u*), with a fixed decay time constant *τ*, effectively setting how fast activity can rise or fall, a constant *u*_0_ describing the initial state (set to zero), and noise, which varies at each time step (random walk), where *η* is a normally distributed random variable *η* = N(0,1), whose amplitude is modulated by *a_η_*. The accumulation process was always simulated in steps of 1 ms.

*Visual inputs.* Visual onsets translate each into an automatic transient excitatory input *I^exo^* with maximum intensity *a_exo_* at *t* = *t_onset_* + *δ_exo_* and decrease with time according to the following equation

$$\tau_{\mathrm{on}}\frac{{\mathrm{dI}}_{i}^{\mathrm{exo}}(t)}{\mathrm{dt}}=-I_{i}^{\mathrm{exo}}(t)+a_{\mathrm{exo}}$$

with *τ_on_* defining the transience of the signals. Left (right) stimuli activate the left (right) node specifically.

*Top-down inputs.* Selective signals are modelled as excitatory sustained signals: *I^endo^* = *a_endo_*. In the 200N model, excitatory inputs at the fixation nodes keeps the noisy activity under control until a peripheral stimulus is detected. In the 2-node version, this is replaced by top-down inhibition during the fixation period (depicted in Fig. 4 as the blue inhibitory connectors from endogenous control). Thus *I^endo^* switches from inhibition (*a_endo-fix_*) during fixation, to excitation (*a_endo_*) at target location when a target is detected (at *t* = *t_onset_* + *δ_endo_*). This feature is an over-simplification and prevents the model from capturing the early part of the saccadic distribution (fast shoulder, see Fig. 8). Note that ‘top-down' or ‘endogenous' signals are underspecified or mysterious in all models, in the sense that we simply do not know how the brain translates the instructions delivered by the experimenter into selective signals biasing activity in favour or against a response option. In some models, these endogenous or goal-related biases are less explicit, being captured by differential mean drift rates or input strengths. Although we have very explicit endogenous inputs here, they do uncontroversial jobs that we know occur in the brain: inhibition to limit anticipatory responses (a keep-still signal akin to fixation activity in saccade models) and selective enhancement of the target signals. The purpose here is not to question how this occurs.

*Output time and variance.* Saccade latency is the time that threshold is reached plus an output delay *δ_out_*, consisting of a fixed delay followed by a random value drawn from a gamma distribution with scale *k* and shape *λ* (following conclusions from Section 3.1). The scale was 0 in the saccadic condition, effectively reducing output time to the constant value suggested by electrophysiological recordings, as in previous models (see Section 1.1.1).

**Fig. 5:**
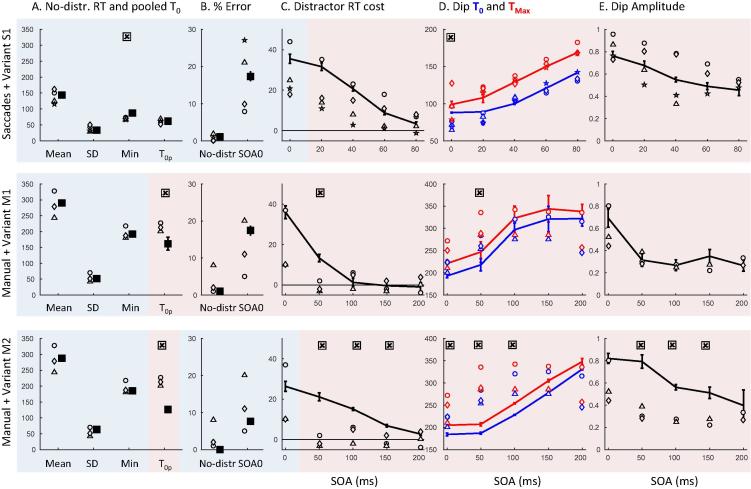
Individual estimates from previous saccadic work and Experiment 1 (empty circle, triangle, diamond and star for Obs. 1–4) along with simulated estimates from variants S1, M1 and M2 (full squares and lines, error bars are SD from 10 independent simulations). Blue shaded areas indicate those estimates used to constrain model parameters, while red shaded areas show the model predictions (i.e. indicate values that were not taken into account to fit the model). Crosses indicate those estimates where model simulations fall outside the range of observed values. S1 lacks very short responses (fast shoulder, see Section 4.2 and Fig. 8), leading to an overestimation of min RT and *T*_0_ at SOA 0, but captures well the pattern of saccadic data otherwise. Variant M1 is simply S1 + extra output time (constrained from the whole baseline RT distributions; mean, SD and Min are shaded in blue as a proxy). M1 overestimates RT cost at SOA 50 and underestimates dip timing, but its predictions are otherwise quite good considering nothing was adjusted except output time. Variant M2 overestimates RT costs and dip amplitudes at multiple SOAs and underestimates dip timing. Note that only dips with amplitudes larger than 16% were detected and therefore contributed to mean dip amplitude and timing estimates from the models. (For interpretation of the references to colour in this figure legend, the reader is referred to the web version of this article.)

*Constraining parameters.* The model contains 16 parameters (see Table 2) To start with, we let 6 vary to account for the saccadic data (parameter set S1) and 9 vary for manual data (M1 and M2); 3 of these were common between modalities and 6 could differ. The other parameters were inherited from the previously published version of the 200N DINASAUR model, and constant between modalities. Critically none of these parameters were allowed to vary between SOAs (SOA affects only the distractor arrival time). The numerous sources of non-linearity introduced in DINASAUR (the model has no analytical solutions) and the time taken by simulations make it currently impossible for us to perform formal fitting (see Section 8.5. Limitations). Instead, we adopted an iterative, simulation-driven approach to constrain our free parameters in a sequential hypothesis-driven manner, in order to simply find whether the model is *sufficient* to capture the patterns of data (even if the parameters we use are not the exact optimal settings, nor necessarily the unique ones). We used the following sequence:

(1) We started by adjusting the 6 free parameters for the saccade condition. The *Act*, *Inh*, *a_η_* and *a_endo-fix_* were first adjusted together to ensure neuronal activity had a satisfying balance of stability (preventing noise-related anticipatory responses in the absence of inputs) and reactivity (saccades normally triggered to salient visual inputs). The saccadic *a_exo_* and *a_endo_* were then constrained from the summary statistics for the baseline and SOA 0 conditions (variant S1, Fig. 5).
(2) We then attempted to account for the extra delay and variance of manual responses compared to saccades (Section 4.3) using two extreme hypotheses differing from S1 either exclusively in the motor output time (M1, using three parameters *δ_out_*, *λ*, *k*) or mainly in the decision time (M2, using parameters *a_exo_*, *a_endo_*, *a_η_*).
(3) Last, we tested the effect of adjusting mutual inhibition (*Inh*).

### 4.3 Constraining non-decision time with 2N-DINASAUR

*Rationale from saccades.* A strong conclusion resulting from the study of saccadic dips in Bompas and Sumner (2011) was that the onset time of the dip in the latency distribution (*T*_0_) gives a direct estimate of the non-decision time – that portion of response latency that is not accounted for by the action selection process – within the framework of our competitive accumulator model. Non-decision time is a sum of input (delay due to sensory processing *δ_vis_*) and output times (or post-decisional delay *δ_out_*). Therefore, providing *δ_vis_* and *δ_out_* are constants and enough trials are available to estimate *T*_0_:

$$T_{0}=\text{SOA}+\delta_{\mathrm{vis}}+\delta_{\mathrm{out}}$$

Note that *δ_vis_* is by default assumed to be equal for targets and distractors since these are non-selective signals (if these were to differ, *T*_0_ would reflect the input delay of distractors). The reason the dip onset does not involve any decision time is because the very start of the inhibition effect represents the case where the distractor signal arrives (*δ_vis_* after its onset, i.e. SOA + *δ_vis_* after target onset) at the selection system just before the decision threshold is reached by the target activity (i.e. *δ_out_* before the response would have occurred). This corresponds to the definition of the threshold – the time beyond which the initiation of the response can no longer be delayed. In order to test this logic, Supplementary Fig. 3 shows a series of simulations varying multiple parameters that influence the decision time but not the non-decision time. The simulations show that none of these parameters affect *T*_0_.

The time taken for a saccadic decision to become an executed saccade is known (*δ_out_* about 20 ms). Thus with a dip onset latency of 70 ms for saccades, it follows that visual signals in our experimental design can reach selection processes in 50 ms, while mean decision time should be around 70 ms in order to produce a mean saccadic latency of 140 ms. Note that for extracting decision and non-decision times, the peak of the distraction ratio (*T_Max_*) is actually less theoretically meaningful than its onset, as the peak will depend on both timing and amplitude (bigger effects have later peaks, because the onset does not change, see Bompas & Sumner, 2011, for discussion). The only occasions when dip onset may not exactly reflect non-decision time is when non-decision time + SOA coincides with a time bin within the RT distribution that is empty or does not contain enough trials, for instance at the very beginning or the end of the RT distribution, or if the study is underpowered. Note that smoothing helps in getting robust estimates of *T*_0_ but anticipates them in a fairly systematic manner (present smoothing means that 7 ms should be added to all *T*_0_ estimates).

There is, of course, likely to be some variability in sensory and output delays even in saccadic decisions, but their contribution to the overall variance is considered to be relatively small (Munoz and Wurtz, 1993, Smit and van Gisbergen, 1989) and a small amount of variance in non-decision delays hardly affects the properties of the model. Thus, for simplicity, this variability is ignored in the present simulations of saccadic behaviour and only considered to account for the larger variance of manual responses compared to saccadic responses.

*Non-decision and decision times for manual responses.* The same logic of extracting non-decisional delays from *T*_0_ should hold for manual responses, irrespective of the specific model chosen, as far as it assumes:

(i) The existence of automatic inputs to the decision process, where the onsets of targets and distractors produce bursts of similar amplitudes;
(ii) Mutual inhibition between alternative action plans (see Section 1.1.3).

However, inferring non-decision time from *T*_0_ is slightly less straightforward if non-decision times are allowed to vary across trials: *T*_0_ would then tend towards the *minimum* value for non-decision time, but a very high number of trials would be required to observe this minimum. With sample sizes around 500–1000 trials per condition and non-decision times following a gamma distribution with shape 3 and scale 23, our model simulations show that *T*_0_ falls in between the minimum non-decision time and its mean.

Another limitation is that the onset time of the distraction effect for manual responses can only be approximately estimated from Experiment 1, because interference is only clear at SOA 0 but not at SOA 50 or beyond. Thus the earliest interference coincides with the beginning of the RT distribution, where it is hard to measure. The same limitation occurs for saccades at SOA 0, but since longer SOAs also show dips for saccades, pooling these SOAs provides robust estimates of dip timing overall. In contrast, pooling is not clearly helpful for manual responses. Although some manual responses are performed with RT before the dip, these are partly anticipations (equally likely to be correct or incorrect) and are therefore not helpful to reveal dip onset because they are not visually driven. If our logic is right though, interference effects should also be present at intermediate SOAs (between 0 and 50 ms) and locked on distractor onsets. This hypothesis is tested (and verified) in Experiment 2.

Despite these limitations, it is already clear that it is much later than for saccades. There was no sign of it in the window where it is seen for saccades (starting 70 ms post-distractor); instead it starts on average around 200 ms after the distractor (Table 1, Fig.2E–F and Supplementary Fig. 2B). Thus, our logic would suggest that manual mean *non-decision* time (*δ_vis_* + *δ_out_*) must be higher than 200 ms. This further suggests that mean *decision* time must be below 80 ms (since mean RT is 280 ms) and could then be similar to saccades (70 ms). This possibility is investigated in model variant M1, which is simply model S1 with added motor output time following a gamma distribution (following Section 3.1). The predictions for M1 are overall good, except for the RT cost and dip timings at SOA 50 (see Fig. 3 column 4 and Fig. 5 row 2).

If, on the other hand, input and output delays were similar to saccades, and the extra latency of responses was entirely due to a longer decision process, we would expect the inhibition effect of distractors to occur at about 70–90 ms for both modalities, and we would get dips in the manual distribution even for distractors 100 or 200 ms after the target. Since this extreme scenario is already incompatible with evidence of longer output time for manual responses (see introduction), we implemented a more moderate scenario, variant M2 (Fig. 3, Fig. 5), where manual output time is taken from intracranial recordings (Miller et al., 2009), and thus the extra RT for manual responses is shared between non-decision and decision time: output time was 85 ms and had no variance, providing a total non-decision time of 135 ms, while for the decision process, we decreased exogenous and endogenous signals and decision noise (note that *increasing* noise would instead speed up RT and thus decrease RT variance, as it helps subthreshold activity reach threshold, see Supplementary Fig. 3). Figs. 3 (right column) and 5 (bottom line) show that this hypothesis predicts clear dips at SOA 50 and beyond. Predicted amplitudes are much higher and onsets earlier than observed in the data for these SOAs. M2 thus performs less well than M1, despite more information being fed into fitting its parameters than M1 (blue shaded areas in Fig. 5). Indeed, M1 simply uses the baseline manual distribution to constrain the motor output time (parameters of the gamma distribution), all the other parameters being inherited from S1. M2 additionally uses the error and RT costs at SOA 0 in an attempt to predict behaviour at longer SOAs.

Note that any other ways of increasing decision time and variance (such as increasing threshold or introducing variance in the strength of exogenous or endogenous signals) will have the same effect, as it will necessarily increase the frequency of slow decision times, which are subject to distractor interference at large SOAs. All would result in underestimated dip timing at short SOAs and clear dips even at long SOAs, which is clearly not the case in the data.

Since variant M1 provides a reasonably good fit to the data, while variant M2 is worse (or no better) in every respect, we conclude that longer decision time is unlikely to be a major part of the reason manual responses are longer than saccadic responses. Instead, most of the difference in mean RT and variance likely originates from non-decision time. Furthermore, if we make the sensible assumption that extra time and extra variance are coupled, i.e. that the extra variance occurs at the same stage as the extra delay, our results also suggest that the variability associated with the decision process is similar in saccades and manual responses.

*Sensory or motor noise?* For the extra variance associated with manual responses to occur during non-decisional time, it must take place either at the pre-decisional (sensory) or post-decisional (motor) periods. Note that only the location of the *variance*, not that of the extra delay, affects the simulation outcomes. Furthermore, when testing the effects of adding sensory noise before the decision process, an important aspect is whether this variance is independent or yoked between the two nodes. While it is not normally included in models of this type, yoked noise is likely to occur (Bompas, Sumner, Muthumumaraswamy, Singh, & Gilchrist, 2015) and could represent, for example, general changes in arousal or the oscillatory dynamics of the visual system from trial to trial. From the point of view of behaviour modelling, the effect of yoked noise in the sensory delay is indistinguishable from that of motor noise (given we have stable, not ramping, baseline activity). However, adding independent sensory noise can have specific effects, in particular when it comes to generalisation from SOA 0 to longer SOAs. Adding the extra variability needed for manual responses as independent sensory noise reduces the RT cost at SOA 0, because the optimal interference occurs when target and distractor signals arrive together into the decision process, and sensory noise reduces the number of trials when this happens. At the same time, RT cost at SOA 50 remains high because, on the proportion of trials in which the sensory delay for the distractor is shorter than that for the target, the effective SOA at the decision process is now closer to zero, producing a strong distractor effect. Thus adding independent sensory noise takes us in the direction of making the distraction effects more similar for SOA 0 and SOA 50, which takes us away from the pattern of observed manual behaviour.

In conclusion, splitting the extra non-decision time into extra sensory delay and extra motor output time (Miller et al., 2009) is certainly plausible given the different pathways through the brain that feed saccadic and manual motor areas, but, from the point of view of the model, this is indistinguishable from keeping sensory delay identical (at 50 ms as for saccades) and extending output time. If extra sensory delay is accompanied by extra variance, this is likely to be mainly yoked between stimuli, because independent sensory noise makes the model less able to capture the data (see also (Bompas et al., 2015) for evidence for yoked noise). Since yoked sensory noise would be indistinguishable from motor noise in our simulations, we also put all the extra noise in the output time (see Table 2, Table 4).

## 5 Experiment 2: distraction effects at intermediate SOAs

### 5.1 Rationale and predictions for SOAs 20 and 40 ms

Above we found that variant M1 captures reasonably well manual patterns of data simply by adding non-decision delay and variance to the model variant capturing saccades (S1). However, Experiment 1 is insufficient to constrain the manual variant. Observed dip timing from Experiment 1 could be inaccurate, since interference was only clearly visible at SOA 0, when the dip onset is often confounded with the beginning of the RT distribution. Moreover, one key property of saccadic dips is that they are time-locked to distractor onset. Thus, if our logic is correct and interference in the manual modality reflects the same process as in the saccadic modality, we predict that dips should be obtained at intermediate SOAs (20 and 40 ms), and that their timing should increase and their amplitude decrease as SOA increases.

Moreover, in Experiment 1, there was a trend for interference effect at SOA 0 to be smaller for manual responses than for saccades, but large individual differences made this trend unclear. Furthermore, the use of different SOA ranges and distractor probability between experiments could have affected the interference effect (Wagenmakers, Ratcliff, Gomez, & McKoon, 2008). Therefore, in Experiment 2, we used the same participants and same SOAs for both manual and saccadic versions of the task in alternating blocks randomised across participants.

### 5.2 Methods

Four new observers participated. Observer 1 was author CH, while Observers 2–4 participated for a small monetary payment. All had normal vision. All aspects of the stimuli, procedure and analysis were identical to those described for Experiment 1, except that we used SOAs of 0, 20 and 40 ms in both manual and saccadic versions of the task. Saccadic and manual blocks were interleaved, with two subjects starting with the manual condition and two subjects starting with the saccadic condition. Each participant performed 8 blocks of each task (480 trials per block), with an equal number of trials in each condition. This represents 480 trials per condition in total, and thus 960 trials for each distractor condition after pooling left and right target trials. Distractors were present in 75% of trials. Saccadic reaction times were extracted according to Bompas and Sumner (2011).

### 5.3 Result overview from Experiment 2: SOA 0–40 ms

**Fig. 6:**
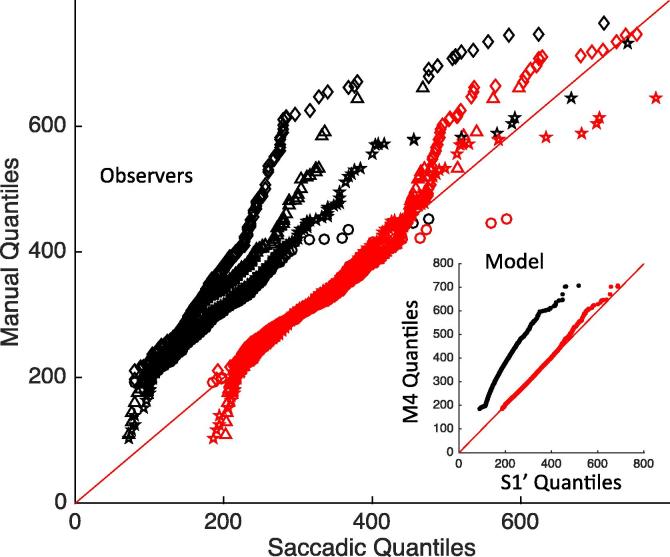
Q-Q plot using individual data from Experiment 2 (empty circle, triangle, diamond and star for Obs. 1–4). Black points: original data. Red points: gamma noise added to the saccadic data. Red line is the identity line. Inset: Simulated data (see Section 6). (For interpretation of the references to colour in this figure legend, the reader is referred to the web version of this article.)

**Fig. 7:**
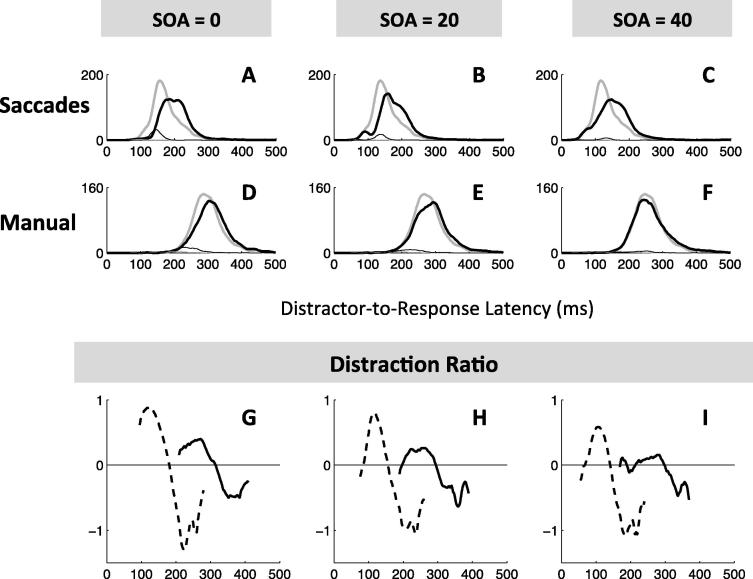
Effects of distractors on saccadic and manual reaction times locked on distractor onset and pooled across 4 subjects for SOA 0, 20 and 40 ms (same convention as in Fig. 2). (A–F) Grey lines: no distractor condition; black lines: distractor present; thick lines: correct responses; thin lines: errors. (G–I) Dashed: saccades; Full: manual.

**Fig. 8:**
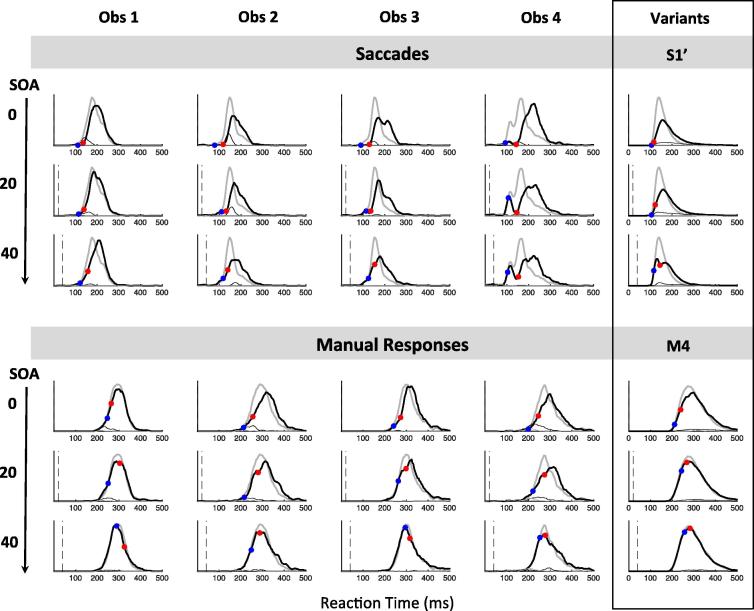
Results of Experiment 2 for each observer, along with model simulations (same conventions as in Fig. 3; grey lines show no distractor condition; black lines show distractor present; thick lines are correct responses; thin lines are errors; blue and red dots indicate dip onset and maxima). Interference from distractors was visible for each participant at all SOAs and for both modalities. Models S1′ and M4 are explained in Section 6. (For interpretation of the references to colour in this figure legend, the reader is referred to the web version of this article.)

Table 3 and Fig. 6, Fig. 7 summarise the key aspects of the observed data, including baseline distributions and interference effects. Fig. 8 shows individual data.

### 5.4 Baseline RTs across modalities

As for Experiment 1, we characterised the delay distribution that should be added to baseline saccadic RTs to make them similar to baseline manual RTs, this time for each participant separately. All participants were slower in the manual condition and their skew was reduced (Table 3), while only two were clearly more variable. Each type of distribution (uniform, Gaussian or gamma) offered a similarly good match for observers 1, 2 and 4, but a gamma distribution offered a much better match for observer 3 (Supplementary Fig. 1). We therefore kept a gamma distribution in our modelling. Individual Q-Q plots (Fig. 6) lead to the same conclusion as from Experiment 1: adding gamma noise very much reduced, but did not eliminate, the difference between modalities. Again, this suggests a small difference in the decision processes between modalities, on top of extra non-decisional delay and noise that accounts for most of the difference.

### 5.5 Distraction effects from Experiment 2

As Fig. 7 shows, interference from distractors were clear at all SOAs for both saccades (KS tests on pooled distributions across observers, all p < 10^−19^) and manual responses (p < 10^−4^). Saccadic results replicate previous findings reported in Bompas and Sumner (2011), showing an overall shift of the latency distribution at SOA 0, followed by clear dips time-locked on distractor onset at SOA 20 and 40 ms. The distraction ratios is positive from 70 ms to 140 ms after distractor onset, which also coincides with an increased occurrence of errors. Manual results also show clear interference effects, not only at SOA 0 as previously shown in Experiment 1, but also at SOA 20 ms and, with a small amplitude, at 40 ms. Distraction ratios is positive from 200 ms to 270 ms on average, similar to Experiment 1. Dips are reduced in amplitude and delayed by 120–160 ms compared to saccades, confirming the conclusions from Experiment 1.

All individual observers (Fig. 8) showed the key effects evident in the pooled plots (Fig. 7): Dips in both modalities, with amplitude and timing both strongly modulated by SOA (all *p* < 0.005) and modality (main effect of modality on max dip ratio, *T*_0_ and *T_Max_*, all *p* < 0.001). Similarly average costs to RT and errors were clearly present for both modalities (paired T-test between baseline and simultaneous distractor condition: all *p* < 0.05), and RT cost was consistently higher for saccades than for manual responses and decreased with SOA (repeated measures two-way ANOVA showed main effects of modality and SOA, both *p* < 0.05, with no significant interaction between them). Error rate increases showed a main effect of SOA (*p* < 0.001) but not modality.

## 6 Modelling results: the balance of input signals

The Q-Q plots in Fig. 1, Fig. 6 indicated that adding gamma-distributed extra output delay accounts for most of the difference in baseline distributions between modalities. Similarly, Fig. 5 showed that much of the data from distractor conditions could also be reasonably well fitted by simply adding this extra output time and variance to the saccade model (while adding extra delay and variance to the decision process instead provided worse fits). However, in both the Q-Q plots of baseline distributions, and in the data from distractor conditions, some important discrepancies remained – especially for distractors at SOA 50. Experiment 2 confirmed the results and logic from previous sections, and also provides the additional data for dip timing and amplitude needed to constrain further adjustments to the model.

As outlined in 1.1.2, manual and saccadic action selection mechanisms are likely to differ in the relative influence of fast automatic and slower selective signals. DINASAUR models produce dips because of the modelled properties of these input signals: the automatic signal from the distractor is sharp and transient, while the endogenous signal favouring the target is sustained. For saccades, the amplitude and sharpness of the automatic distractor signal means that it can transiently overcome mutual inhibition from the target node, and thus influence the decision dynamics even when the target has a head start.

**Fig. 9:**
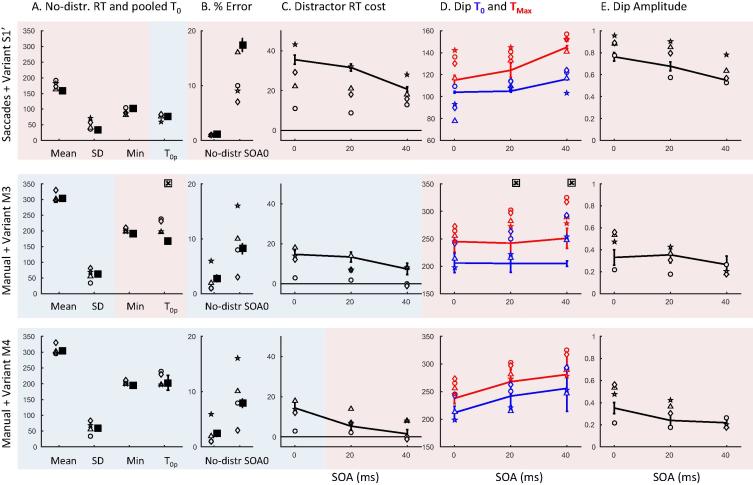
Behavioural estimates from Experiment 2, along with simulated estimates from model variants S1′, M3 and M4. Variant S1′ is simply S1 with adjusted visual delay to match the longer mean dip onset for this cohort (note that no attempt was made to match other aspects of the data). Variant M3 has longer and variable output times (as Variant M1 from Fig. 5), as well as reduced exogenous input compared to S1′ and M1. M4 is the same as M3 except that the delay of exogenous signals is increased to match that of endogenous signals.

The timing of saccadic dips was slightly higher in this new cohort compared to previous work, and was captured by a 16 ms increase in visual delay in our model (variant S1′). No further attempt was made to adjust model parameters to this new cohort, as all estimates remained satisfying (i.e. within the range of natural observer variation, see Fig. 8, Fig. 9). For manual responses, the RT cost and dip amplitudes are reduced, becoming very small by SOA 40, suggesting reduced amplitude and sharpness of exogenous signals. This hypothesis is captured by variant M3 of the model (Table 4, Fig. 9). Reducing signal strength means that the model is more strongly driven by noise, leading to more errors even in the baseline condition. To keep the number of errors equal across modalities, variant M3 also has increased amplitude of endogenous signals to compensate for reduced exogenous input.

Model variant M3 can produce accurate dip amplitudes throughout SOAs but their timings remain underestimated and fail to rise linearly with increasing SOA (Fig. 9 middle panels, red and blue lines). An important point is that, although *T*_0_ is underestimated, we cannot simply increase non-decision time in order to increase *T*_0_, as this would also increase min and mean RT, rending them inaccurate. Furthermore, this would not help the model better capture the relationship between SOA and *T*_0_. Note that dip timing was more strongly affected by SOA in the manual compared to the saccadic condition (modality x SOA interaction effect on *T*_0_ and *T_Max_*, both *p* < 0.05). The reason why this linear rise is strong for manual responses is that manual *T*_0_ tend to be later than the minimum baseline RT (*p* = 0.02 when pooling across experiments) and can therefore be correctly estimated at each SOA. In contrast, saccadic *T*_0_ tend to be earlier than the minimum RT (*p* = 0.015). Consequently, observed dip onsets at short SOAs (0 and 20) tend to be overestimated for saccades (the hypothetical onset falls too early within the distribution to be detected), but not for manual responses. All our model variants so far (S1, S1′, M1, M2, M3) produce *T*_0_ that are shorter than minimum baseline RT (because the minimum decision time is not zero). This captures well saccadic data, but not manual responses.

The pattern of manual data thus suggests that exogenous inputs from distractor – which drive the dip – occur later for manual responses, and closer in time to the endogenous inputs from the target – which drives responses in the baseline condition and thus determines minimum RT. This additional hypothesis is illustrated by variant M4 of the model, where the exogenous delay was increased to match the endogenous delay. Although it remains unclear whether this assumption is realistic, only this adjustment appeared to satisfyingly equalise *T*_0_ and minimum RT, and variant M4 provides excellent behaviour along all our estimates. A complementary option (not illustrated here) would be to assume that exogenous signals from distractors are slower than those from targets, but only for manual responses, not saccades. Such asymmetry in exogenous delay could possibly result from feature-based attentional bias, which would have to be stronger for manual than saccadic responses, though we can only speculate on whether and why this could be expected (for example, due to the different balance of perceptual pathways feeding each system). This further refinement, added on top of variant M4, would produce *T*_0_ *later* than minimum RT, matching the data from observers 1 and 3 in Experiment 2, but is unnecessary for observers 2 and 4.

Variant M4 produces shorter mean decision times for manual responses (56 ms vs 73 for saccades), since both inputs now join forces synchronously to drive accumulation to the threshold. However, further minor tweaking of model parameters can provide equal decision time across modalities while keeping an equally good match on all estimates (variant M5: same as M4 but *Th* = 0.87, *τ_on_* = 10, *a_exo_* = 12, *a_endo_* = 6, *δ_out_* = 142, not illustrated). This variant is equally parsimonious as M4 in terms of comparing saccades to manual responses, simply trading a decrease in sharpness (*τ_on_* increase from S1′ to M4) for an increase in threshold. Importantly, further increases in threshold leading to longer decision times for manual would not capture the data well (see Section 4.3). Our modelling therefore suggests that manual responses have similar, or possibly shorter, decision times than saccades.

We can therefore conclude that, despite an apparent qualitative difference between saccadic and manual behaviour – the presence or absence of a clear bimodality in RT distributions (dips) – the same model architecture can capture both patterns of behaviour with minor quantitative adjustment. This offers the promise of a simple means to generalise between saccadic and manual studies.

## 7 Lateral inhibition and winner-takes-all behaviour

**Fig. 10:**
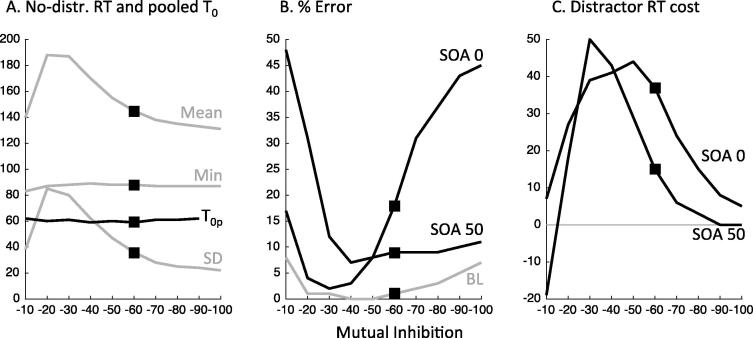
Effect of varying mutual inhibition (*Inh*) on baseline (grey) and distractor condition (black) estimates from the S1 variant. Black full squares indicate the estimates obtained with *Inh* = −60, as used in all variants illustrated in this article. Plot A shows that dip onset and min RT are immune to mutual inhibition values, since mutual inhibition affects the decision process while min RT and dip onset mainly reflect non-decision time (Section 4.3). Plot B shows that too much or too little mutual inhibition allows too many errors in some conditions. Plot C shows that hypothesising reduced *Inh* for manual responses results in an increase, rather than the desired decrease of RT cost, unless *Inh* becomes so small that errors become unrealistically high (Plot B).

There is no a priori reason to assume that lateral inhibition must be the same for saccadic and manual motor competitions. Indeed, as outlined in the introduction, we might expect winner-takes-all behaviour to be stronger for saccades than for manual responses because it is possible to execute more than one manual action simultaneously. However, either increasing or decreasing lateral inhibition in our models does not account for the differences between manual and saccadic response patterns (Fig. 10). In 2N-DINASAUR, reducing mutual inhibition to a very small level would, of course, reduce the effect of late distractors on latency as required by the observed data. But it would also allow too many errors for late distractors (Fig.10B). Increasing mutual inhibition also destroys any inhibition effect for late distractors (Fig.10C), but has other unwanted effects, such as making the model quite unstable and thus creating too many errors for simultaneous distractors (Fig.10B). Mutual inhibition generates winner-takes-all behaviour; thus if the distractor activity is not suppressed then the target node will be, creating error responses. This occurs because fluctuations in the noise can allow the initial distractor activity to rise higher than the target activity, at which point the latter is suppressed and the distractor becomes the all-taking winner. With too much mutual inhibition, fluctuations in the noise alone, even in the absence of distractors, can favour the incorrect response enough to kill the desired response (Fig.10B grey line).

Fig. 10 points out an important property of mutual inhibition: although the inhibition effect depends upon mutual inhibition, it is not simply the case that more mutual inhibition creates a larger effect. It is an inverse-U function. The distractor effect depends on two things: inhibition between the nodes and a period where both nodes are active enough to exert that inhibition. If the distractor node is active for longer time, it has more effect. Therefore a lower level of mutual inhibition can counter-intuitively increase distractor effects (Fig. 10C) because the distractor node is crushed more slowly.

In between the problems created by too little or too much mutual inhibition, there is a range of mutual inhibition strengths that can give satisfying simulations of the data as long as changes to the exogenous and endogenous signals and noise are also made. Thus our modelling does not exclude the possibility of some difference in mutual inhibition between manual and saccadic responses. However, we find that different mutual inhibition is neither necessary nor sufficient to account for the different behavioural patterns we observe.

## 8 General discussion

Our key behavioural result was that manual responses did show an interference effect from task-irrelevant visual distractors, despite previous studies not finding the RDE with manual responses (McIntosh and Buonocore, 2012, Rafal et al., 1990, Sumner et al., 2002). However, this effect is reduced compared to saccades, and becomes hardly distinguishable from noise when distractors follow the target by 50 ms or more.

Relying on a neural field model previously used for saccades, we designed a 2-node version (2N-DINASAUR) to test whether and how it was possible to generalise between modalities. The model was able to capture the patterns of observed behaviour in both modalities with the following conclusions:

(1) The timing of manual and saccadic interference effects (mean RT cost and dips where apparent) relative to their response latency distributions indicate that the two systems have similar decision durations. The overall longer latencies of manual responses are best attributed to extra non-decisional delay and variance (Section 4.3 and Fig. 3, Fig. 8).
(2) The balance and relative timing of signals feeding the decision processes likely differ across modalities: automatic visual signals to manual decisions would be weaker, slower and less sharp, while sustained and selective inputs would be comparatively stronger (Section 6 and Fig. 8, Fig. 9).
(3) Differences in mutual inhibition in 2N-DINASAUR were neither necessary nor sufficient to capture modality differences (Section 7 and Fig. 10).

As in any modelling work, we can only conclude that the model presented is sufficient, rather than necessary (amongst all possible model alternatives), to capture the data. These main conclusions are discussed below along with previous work and alternative accounts.

### 8.1 A manual distractor effect found here but not previously

Previous research has not found the RDE for manual responses, while we found it in two independent experiments. We can suggest three possible reasons for why this might be:

First, since interference effects are reduced in manual responses, more trials than used in some previous studies could be required to distinguish this weak effect from noise (for example the present experiments have about 8 times more trials per condition than Sumner et al., 2002).

Second, since the effect is overall smaller with manual responses and decreases rapidly with SOA, a sub-optimal SOA is more likely to result in not finding the effect. The optimal condition for obtaining interference is when the distractor signals arrive together with the target signals at the action selection stage. This does not necessarily correspond to when distractors are displayed simultaneously with the target, depending on relative sensory delays between target and distractor signals, which would likely differ across studies. Previous work on saccades has showed how features like contrast or colour of the distractor affected the optimal SOA (Bompas and Sumner, 2009a, Bompas and Sumner, 2009b, Born and Kerzel, 2008) and the timing and amplitude of dips (Bompas & Sumner, 2011). Thus, the optimal SOA will depend on the features of the stimuli used as targets and distractors, as well as their respective location in the visual field and, possibly, the attentional requirements of the tasks. For weaker distractor signals in the manual domain, the RDE will be highly sensitive to any departure from this optimal timing.

Last, distractors in some previous literature occurred at irrelevant positions (McIntosh & Buonocore, 2012), i.e. never occurred in a location that was part of the currently possible set of target locations. The uniquely direct mapping between stimuli and saccade responses might allow distractors to cause interference wherever they occur (for a review see Casteau & Vitu, 2012). Still, location relevance of distractors modulates their effects in saccades (Reingold & Stampe, 2004). If the same modulation applies to manual responses, distractors at irrelevant locations would be expected to produce even smaller interference than reported here. Consistent with this speculation, the remote distractor effect is modulated by visual similarity between target and distractor (Born & Kerzel, 2009). Attention also has a strong modulating influence on other automatic interference effects. For example, masked priming effects are modulated by temporal, spatial and feature-based attention – they tend to occur only for primes that are similar in location and form to currently possible targets and presented in the attended temporal window (Eimer and Schlaghecken, 1998, Lachter et al., 2004, Naccache, 2005, Sumner et al., 2006). Likewise, Eriksen flanker effects do not occur when the distractors are too far from the attended target location (Eriksen & Eriksen, 1974).

### 8.2 Why should a model of superior colliculus work for manual decisions?

The original DINASAUR was designed to capture the essential properties of the superior colliculus (Trappenberg et al., 2001), which is a fundamental part of the saccade system. However, the architecture is also inspired by general interactive accumulator models (Kopecz, 1995, Usher and McClelland, 2001) and by the concepts of dual information routes traditional in cognitive psychology, where automatic signals compete with selective signals. Thus DINASAUR is probably best considered not as a model of the SC, but as a general simplified model of a competitive decision map containing the essential properties common to action selection across modalities. That is, an interactive race to threshold fed by a transient automatic signal arising from both targets and distractors, followed by a sustained signal that favours the target, mutual inhibition proportional to node activity, and some form of control to keep the system stable before the stimuli arrive. The essential feature of DINASAUR that allows it to capture both saccadic and manual behaviour is the ability to adapt the balance and timing between the sharp transient and the linear sustained signal. While the importance of visual transients in SC activity has been emphasised (Boehnke & Munoz, 2008), our modelling suggests that these are present but less important for manual responses.

### 8.3 Are manual and saccadic decision times similar?

One of the major conclusions we reached is that, although manual responses are slower and the onset times of the inhibition effect is later than for saccadic responses (200 ms vs 70 ms post-distractor), manual decision time is unlikely to be longer than saccadic decision time.

We began this research with a naive expectation that, if saccadic inhibition occurred at all for manual responses, it should be visible in the RT distribution in a not-too-dissimilar time window (i.e. nearer to 100 ms post-distractor than to 200 ms), and thus the optimal SOA to visualise a dip would be around 100 ms (so that the dip falls around 200 ms, within the main mode of the manual RT distribution). However, considering the issue from the modelling perspective allowed us to realise that, in order for manual responses to show an inhibition effect (a dip) at the same latency after the distractor as saccades do, both the input time for the exogenous signal and the output time from decision to action would have to be similar to saccades. This in turn would mean that since manual latencies are longer and more variable than saccadic ones, the decision process itself would have to be much slower. This extreme scenario is incompatible with electrophysiological evidence showing longer output time for manual than saccadic response. Instead, we presented a possible model that would be more realistic, with only some of the extra delay being accounted by decision time (variant M2). However, even this plausible assumption causes the model to show strong inhibition for late distractors, in stark contrast to the observed data (Fig. 3, Fig. 5). Therefore we concluded that very little, if any, of the extra delay and variance for manual responses compared to saccades is accounted for by the decision process.

On the other hand, if manual decisions are not longer than saccadic decisions, then all, or nearly all, of the difference between saccadic and manual (button press) mean latency must be accounted for in the non-decisional input and output delays. This in turn means that the delay between a distractor and its overt inhibition effect within the RT distribution must be correspondingly longer for button presses, exactly as we see in the data. Indeed, we found that behaviour in the two modalities could be modelled by assuming similar time taken by the manual and saccadic accumulations to threshold (or possibly even shorter if the gap between exogenous and endogenous signals is smaller, as in the best fitting model variant, M4).

Thus the differences in behaviour – longer inhibition delay and lack of inhibition for late distractors – actually point towards similar, not different, decision processes. We are not claiming that the selection mechanisms are exactly the same between modalities. For example, we suggest that a different balance and timing of exogenous and endogenous signals is necessary to account for the data. For other parameters, a change was not necessary or useful, but we are not claiming that they should necessarily be the same across modalities. For instance, mutual inhibition depends on the properties of the ‘options map', and different levels of top-down inhibition might be necessary to maintain stability before the stimuli arrive. However, we consider it noteworthy that the different behavioural patterns are most straightforwardly captured without changing these parameters.

The relatively short decision time for manual responses, compared to the overall response time, is in fact entirely consistent with previous modelling in a different context. Verbruggen and Logan (2009) compared an interactive accumulator with a 2-horse race model (no mutual inhibition) for the ‘stop signal task' in which participants manually respond to targets but are occasionally cued to withhold their responses (the stop cue comes after the target, just as late distractors do). It was found that these rather different modelling approaches could both simulate observed behaviour. Verbruggen and Logan's explanation for this was that the decision process is actually rather short, which minimises the difference between the models relative to the effect of the delay between the target and stop signal (which is of course common between the models). The longer the decision time, the more the difference between these two models would become apparent.

Our conclusions using a rapid action selection task compare interestingly with previous work using a perceptual discrimination task with simpler decision models (Gomez et al., 2015, Ho et al., 2009). In the linear ballistic accumulation model used by Ho et al., the activity corresponding to each response option accumulates independently (without mutual inhibition) and linearly (without diffusion noise) from a start point to a threshold. On each trial, the start point and accumulation rate for each option are randomly drawn from distinct distributions (Brown & Heathcote, 2005). Note that this model is unable to produce dips (Bompas & Sumner, 2011) and was therefore not an option for us here. Ho et al. concluded that mean non-decision time for manual responses (134 ms) was about 80 ms longer than for saccades (53 ms). Gomez et al. used the diffusion model (Ratcliff & Rouder, 1998), in which noisy activity fluctuates between two thresholds, one for each possible response option. This means that evidence in favour of one response is also evidence against the alternative response, as if signals favouring each response were perfectly and negatively correlated. This model is able to produce dips, but unless parameters are allowed to vary across SOAs, it cannot capture manual data (i.e. it cannot produce clear dips at SOA 0 and no dip beyond 50 ms with the same set of parameters; analysis not shown here). Gomez et al. concluded that mean non-decision time for manual responses (327 ms) was also about 80 ms longer than saccades (254 ms), although their estimates are overall much longer than in Ho et al. Our conclusions are qualitatively similar, although our estimates fall in between those previous studies (around 200 for manual and 80 ms for saccades), and we believe fit well with known neurophysiology of sensory delays for visual onsets in different brain areas and motor output times.

Both previous studies also suggested the same values for drift rate mean and variance across modalities (i.e. information flow), consistent with the expectation that perceptual decisions may not be embodied in motor planning processes. Interestingly the studies also suggested a slightly higher (more cautious) threshold for manual than saccades, and indeed we find it plausible that response threshold remains a property of the output system even when the task is considered perceptual. Note that in Ho et al. (2009) and Gomez et al. (2015), the higher thresholds for manual responses would result in slightly longer decision times compared to saccades. In contrast, we concluded there are similar or slightly shorter decision times for manual responses. Our conclusions rely on strong non-linearity in the accumulation process (resulting from both transient and sustained signals). Broadly though, the three studies are consistent in suggesting that the difference in non-decision time between modalities is much larger than any difference in decision time.

Our results and model did not require an increased threshold for manual responses, but such a situation can be accommodated (see the end of Section 6 where model variant M5 is discussed). It may be considered a weakness of DINASAUR that one parameter can be traded against another in this way, so that the data do not constrain a unique solution (see discussion of limitations below). Alternatively, such interplay between parameters helps us develop our understanding of models and what to look out for in data. In simpler models it is possible that differences in data that do not actually correspond to our conceptual understanding of threshold and caution are captured by differences in threshold because other parameters are absent. Thus we believe it is useful to understand how both simple and more complex models would capture the data, and which parameters can be traded against which.

### 8.4 Modality-specific versus amodal decision area?

An interesting question is whether the choice of similar parameter values across modalities (in our work or in Ho et al.’s) points towards a common decision area or simply reflects similarities across multiple neural networks involved in action selection across the brain. Ho et al. (2009) identified a region (the right insula) whose BOLD activity during perceptual decisions displayed a time course compatible with evidence accumulation irrespective of the modality used to respond. Other brain areas were also identified in which signal increases were consistent with evidence accumulation specifically for saccades (bilateral IPS) or manual responses (contralateral central sulcus). These findings raise the possibility that activity in modality specific areas are actually fed by signals originating from an amodal decision area.

Our modelling also suggests that all core properties of the decision network (firing rate function, decay time constant, self-excitation, mutual inhibition, noise) see Table 2) do not need to differ across modalities to capture the pattern of results. However, in contrast to Ho et al. (2009), the key differences that our design and model allowed us to identify are the relative balance of exogenous and endogenous signals. Similarly, previous work has shown that saccades and manual responses rely on a different balance of fast magnocellular and slower chromatic pathways (Bompas & Sumner, 2008). These results appear at first difficult to reconcile with a single decision area scenario, unless we consider the interesting possibility that the nature, strength and delay of inputs to this area are modulated by the instruction to respond manually or via a saccade.

More likely, different types of task draw differentially on multiple areas capable of supporting decisions. We argued in the introduction that rapid motor tasks are the most likely to draw on decision processes embedded in motor planning networks, and thus be most likely to show differences between modalities. Where the rate limiting decision is more perceptual or cognitive, this might preferentially draw on unimodal areas who then pass on the decision dynamics to modality specific areas as suggested in Ho et al.'s BOLD data.

### 8.5 From differences across effectors to differences across sensory modalities

Consistent with the conclusions of several previous studies, we conclude that differences across effectors mainly reflect differences in motor output and the properties (here strength and timing) of the sensory input to effector-specific selection areas, while the decision stage seems to share the same principles across effectors (Logan & Irwin, 2000). The specific differences we find between sensory inputs will be specific to the visual modality, which we have focused on because it is basis of the vast majority of psychological and neuroscientific studies. Tactile stimuli on the digits, for example, would have less direct input to the saccade system and relatively more direct association with manual actions. Future work may investigate other sensory-effector combinations in the context of distractor interference. The critical point is that sensory input dynamics are an essential part of understanding decision processes.

### 8.6 Limitations

Overall, DINASAUR has more parameters than can be uniquely constrained with our behavioural data. This is because it is partly inspired by the behaviour of neural networks, rather than solely by human overt responses. As a consequence of this complexity, an apparent limitation of our approach is that a good fit does not rule out an alternative hypothesis: in principle, the more complex the model, the more possible a good but biologically meaningless fit becomes. However, it is an essential aspect of our results that no complex or opaque adjustment of multiple parameters was required to capture both saccadic and manual behaviour. Rather, simple hypothesis-driven adjustments sufficed, while many of the parameters inspired by the neurophysiology remained fixed.

This being said, the exact way in which the free parameters are adjusted would undoubtedly benefit from more formal fitting procedures. Unfortunately, these procedures are not yet available to us when using such highly non-linear models to address such dynamic phenomenon as distractor interference. A first reason for this is that, although 2N-DINASAUR is simpler than 200N-DINSAUR, it has almost as many free parameters and, crucially, still no analytical solutions. Secondly, in contrast to previous modelling work that successfully relied on formal fitting procedures (Turner et al., 2013, Usher and McClelland, 2001), our reasoning is based on the *shape of the latency distribution*, not on mean values. Furthermore, the specific comparison across modalities focuses on how this shape evolves as a function of SOA. This means that each step of the fitting algorithm requires the simulation of 50.000 trials (10.000 × 5 SOAs to simulate the data from Experiment 1), with as many different *noise* samplings. On a Mac book pro with 2.9 GHz Intel Core i5, simulating these 50.000 trials takes 3 min. With 9 free parameters in the 1st modelling section, and another 2 added at the 2nd modelling stage, performing formal model fitting would be very expensive in time and computational power. Ultimately, we believe our conclusions would not change if we were to have an algorithm blindly explore the parameter space, but this is a belief based on reasoning and experience of what each parameter does to latency distributions, rather than a conclusion proven by exhaustive exploration of the entire parameter space. To our eyes, models are useful not only when they can be unambiguously ruled in or out by data, but also as tools to understand the potential consequences of different scenarios.

### 8.7 Alternative accounts

There are of course other models (theoretical or computational) that can capture behaviour in one modality or the other. Importantly though, for any accumulator model to spontaneously generate (rather than merely fit data *a posteriori*) the interference effects described in this article in any modality, some ingredients are necessary: some form of automatic signal arising from both target and distractor in order to create interference and errors, and top-down or selective signals/bias to make sure the target wins most of the time despite those automatic signals.

*Mutual inhibition.* A third key ingredient is some form of inhibition between distractor and target. In our model, mutual (i.e. direct and reciprocal) inhibition not only explains the latency increase in the presence of a distractor, but also makes it more likely that only one location can reach threshold at a time, i.e. it implements a winner-takes-all behaviour. However, this property has been recently challenged. First, recent saccadic literature has shown that inhibitory connections are not longer-range than excitatory connections within the intermediate layers of the SC (Isa, 2002, Phongphanphanee et al., 2014), one of the key saccadic decision areas and the inspiration for DINASAUR models (Dorris et al., 2007, Munoz and Istvan, 1998, Trappenberg et al., 2001). Second, winner-takes-all behaviour is not always necessary and can be undesirable when modelling saccade landing position in the presence of multiple neighbouring stimuli within one hemisphere (the “global effect”).

This debate is mostly irrelevant to 2N-DINASAUR, which does not have spatial layout. However, we will take this opportunity to address this important debate in relation to 200N-DINASAUR. Importantly, the above arguments only question *intra-hemispheric* connections, and not mutual inhibition *between* the left and right colliculi (respectively coding for rightward and leftward saccades, refs), nor between the rostral and caudal parts of the SC (coding for fixation and movement), for which there is strong evidence (Takahashi, Sugiuchi, & Shinoda, 2007). We therefore conclude that 200N-DINASAUR relies on safe assumptions for modelling fixation-distal interactions (such as the effect of stimuli at fixation, and gap effect), as well as interactions between left and right targets along the medial line. This is particularly true when focusing on the temporal dynamics of interference (rather than spatial effects), which is the context we have used it in so far.

Apart from mutual inhibition, several other inhibitory mechanisms could mediate distractor interference. One is feedforward inhibition (Purcell et al.), where the visual signal elicited by the distractor (target) is thought to directly inhibit the motor node corresponding to the target (resp. distractor). Note that the classic diffusion model has feedforward inhibition in the sense that evidence for one response is direct evidence against the other. Feedforward inhibition predicts that late distractors will exert a similar interference as early distractors, resulting in strong dips at large SOAs. This is because, in contrast to our model, the inhibition applied on the distractor node from the target node does not grow with the accumulating target activity, and thus with increasing SOA. Such a model could then possibly capture saccadic interference effects, but not manual ones, unless additional specific post hoc assumptions are made (such as allowing visual activity or the strength of feedforward inhibition to vary with SOA, in a modality-specific manner; note that in our modelling we never allowed parameters to vary with SOA).

Another account of the distractor effect (for saccades only) is the “fixation gating” mechanism, which relies on inhibition via omnipause neurons (Casteau & Vitu, 2012). Visual activity in the SC map within 10 deg around fixation has an excitatory effect on omnipause neurons, which in turn inhibit burst neurons within the SC (Yoshida, Iwamoto, Chimoto, & Shimazu, 1999). Thus, the extra visual activity from the remote distractor could indirectly delay saccade execution without decreasing target-related activity. A model precisely implementing this highly plausible idea remains to be developed before its predictions can be discussed. It is unclear for now whether it has any equivalent for manual responses and thus whether it could allow generalisation between saccades and manual responses.

## 9 Conclusion

Our main conclusion is that, for rapid action decisions in a very simple task, where the rate-limiting step is likely to be response selection, the decision mechanisms for manual and saccadic modalities share similar properties, have similar decision time, and should benefit from being investigated within a unified framework. Both modalities can be captured using the same model architecture with only changes to the properties (timing and strength) of the inputs feeding action selection. The same core principles therefore generalise across modalities, but separate action selection processes appear most likely, each receiving different combinations of exogenous and endogenous inputs with specific timings. Since simple tasks ought to be most dependent on modality-specific processes, the conclusion ought to generalise to more complex tasks where the rate limiting steps become more perceptual or cognitive and the differences between modalities should become even less important.

## Figures and Tables

**Table 1:** Individual estimates from previous saccade data (Bompas and Sumner, 2011) and manual data from a new group of participant in Experiment 1. *T*_0_*_p_* is the dip onset estimated from pooled distribution across all conditions, locked on distractor onset. Time estimates (mean and min RT, its standard deviation SD and *T*_0_*_p_*) are expressed in ms.

Measure		Saccades (previous work)				Manual (Exp. 1)		
		Obs 1	Obs 2	Obs 3	Obs 4	Obs 1	Obs 2	Obs 3
Baseline (no distr.)	Mean RT	151	125	163	115	328	244	279
	SD	42	30	50	31	70	41	52
	Skew	3.1	1.9	3.1	2.1	1.7	0.6	1.8
	Min RT	73	66	71	67	217	180	188
Distractor SOA 0	RT cost	43	25	18	20	37	10	10
	% Error	8	21	10	27	5	20	11
All SOAs	T_0p_	59	68	53	62	227	201	214

**Table 2:** Parameters used in 2N-DINASAUR and values used in the simulations illustrated in Fig. 3, Fig. 5. Grey cells indicate those parameters that were fixed (same as 200N-DINASAUR from Bompas and Sumner, 2011). White cells correspond to free parameters. Only 6 of these free parameters were allowed to vary between modalities, 3 of which relate to motor output time (*δ_out_*, *λ* and *k*) and 3 influencing the decision process (*a_exo_*, *a_endo_*, *a_η_*). Note that the SD of the motor output time (given in brackets) is not a separate free parameter, but arises directly from the gamma noise parameters.

**Table 3:** Estimates from Experiment 2 in the saccadic (S) and manual (M) blocks of the task.

		Obs1		Obs2		Obs3		Obs4	
		S	M	S	M	S	M	S	M
Baseline	Mean RT	191	296	163	303	171	330	181	297
	SD	37	35	40	55	55	82	64	70
	Skew	1.3	0.5	1.7	1.6	4.5	2.7	2.4	1.6
	Min RT	105	209	81	199	89	210	87	199
Distractor SOA 0	RT cost	11	3	23	19	30	12	43	13
	% Error	10	8	16	9	6	3	8	16
All SOAs	T_0p_	80	238	77	197	84	231	61	198

**Table 4:** Parameters of saccadic and manual models following Experiment 2. Model S1′ is the same model as S1, except *δ_vis_* was adjusted to reflect saccadic T_0_ in this new cohort (the time difference with *δ_endo_* is kept at 25, leading to 91 in S1′). M3 differs from S1′ in two aspects: motor output time and input strength and transience. M4 only differ from M3 in the timing of exogenous signals.
