# Supplementary material for: Speeded saccadic and manual visuo-motor decisions: Distinct processes but same principles
Source: Cogn Psychol. 2017 May;94:26–52. doi: 10.1016/j.cogpsych.2017.02.002 (PMC5388195; doi:10.1016/j.cogpsych.2017.02.002)
Supplement: Supplementary Figs. 1-3 [file mmc1.docx]

**Supplementary Figures**

Supplementary Figure 1. Comparison of baseline RT in manual and saccadic modalities. KS statistics was calculated between baseline manual RT and baseline saccadic RT with added noise across a wide range of parameters. Only minimum KS statistics are shown here. Ten independent repetitions were run for each dataset to identify the optimal parameters for each of 3 noise distributions: a uniform distribution (pink), a Gaussian distribution (blue) and a gamma distribution (red). This analysis was performed on the pooled data from all participants in experiment 1 (manual RT) and previous study (saccadic RT), represented as squares, as well as for each participant in Experiment 2 (same convention as figure 9). In Experiment 1, adding a gamma distribution to saccadic RT offers the closest match to manual RT, resulting in smaller KS statistics across all repetitions. In Experiment 2, the choice of the noise distribution seems mostly unimportant except for Observer 3, for whom a highly skewed gamma distribution offered a much better match.

Supplementary Figure 2. Individual distraction ratios with 95% confidence intervals from bootstrapping. Ratios are locked on distractor appearance and shown for simultaneous (grey lines) and late (black lines) distractors, for the 4 observers who performed saccades in previous work (A) and the 3 observers who performed manual responses in Experiment 1 (B). Confidence intervals (thin dashed lines) are Bonferonni corrected across the 126 bins used to calculate the ratios. The blue vertical lines indicate the timing of the dip onset as estimated individually from pooling across all SOAs. Interference from visual distractors (departure from zero of the ratio) is clear on all the saccadic data, as well as all the manual data at SOA 0. Manual responses also seem to show an interference effect for late SOAs on observers 2 and 3.

Supplementary Figure 3. Effect of varying model parameters one by one, compared to those chosen in variant S1 (middle row, framed in black). Grey lines show the no distractor condition. Black lines show the distractor condition at SOA = 50ms. See Table 2 for a description of each parameter. Input noise (column F) refers to adding trial-to-trial Gaussian noise to the amplitude of the exogenous (top line) or endogenous (bottom line) inputs. The reference, used in S1, is no input noise.
